# Intraprostatic steroid hormones and endocrine disruptors in prostate cancer

**DOI:** 10.1530/ERC-26-0153

**Published:** 2026-07-07

**Authors:** Jana Vitku, Tereza Skodova, Anezka Varausova, Lukas Gadus, Michal Horenitzky, Marie Novakova, Martin Hill, Michaela Svojtkova, Lucie Kolatorova, Adela Lukaskova, Jiri Heracek

**Affiliations:** ^1^ Department of Steroids and Proteofactors, Institute of Endocrinology, Prague, Czech Republic; ^2^ Department of Urology, First Faculty of Medicine, Charles University, Prague, Czech Republic; ^3^ Department of Urology, Military University Hospital, Prague, Czech Republic; ^4^ Department of Pathology, Military University Hospital, Prague, Czech Republic

**Keywords:** prostate cancer, endocrine disruptor, endocrine-disrupting chemical, steroid hormone, LC-MS/MS

## Abstract

Prostate cancer (PCa) is a hormone-dependent malignancy governed by steroid signaling. Despite characterization of androgen pathways, the broader intraprostatic steroid environment remains underexplored. Endocrine-disrupting chemicals (EDCs) may modulate these processes, yet their tissue distribution is largely unknown. We aimed to comprehensively profile intraprostatic steroids and EDCs and assess their associations with tumor aggressiveness, prognosis, and risk stratification. Thirty steroids and 16 EDCs were quantified using liquid chromatography–tandem mass spectrometry in cancerous tissue, non-cancerous tissues (from peripheral and transition zones), and plasma from 173 patients with localized PCa undergoing robot-assisted radical prostatectomy. Analysis of variance was used to compare analyte levels across biological matrices and according to disease aggressiveness, multivariate statistical models evaluated associations with patient prognosis and risk categories, and machine learning (ML) models were applied for risk stratification. Steroid profiles differed markedly between plasma and prostate tissue, whereas EDCs accumulated in the prostate and were generally not associated with plasma concentrations. Cancerous tissue showed lower conjugated dehydroepiandrosterone (DHEA) and higher pregnenolone, corticoids, and testosterone compared to non-cancerous tissue. Elevated intratumoral cortisol, cortisone, androstenedione, conjugated testosterone, 7-ketoDHEA, and estrone were associated with greater tumor aggressiveness. Higher intratumoral parabens correlated with increased postoperative PSA, while conjugated dihydrotestosterone (DHT) and phytoestrogen daidzein were inversely associated with PSA levels. Higher EDC burden and intratumoral steroid levels were associated with higher risk categories. In contrast, DHT showed a consistent protective profile, being negatively associated with both prognosis and risk classification. Integrating steroids and EDCs improved ML-based risk classification beyond preoperative clinical variables.

## Introduction

Steroid hormones play a central role in the development of the prostate gland and in maintaining physiological homeostasis in the adult prostate, primarily through androgen- and estrogen-mediated regulation (1, 2). Androgens have also long been recognized as key drivers of prostate cancer (PCa); however, growing evidence suggests that additional steroid hormones, including estrogens and progesterone, can contribute not only to tumor initiation but also to disease progression (3).

Accurate evaluation of the biological role of steroid hormones in the prostate requires direct assessment of intraprostatic steroid concentrations, as these more precisely reflect the local tumor microenvironment than circulating levels. However, only a limited number of case–control studies have examined intraprostatic androgen concentrations in treatment-naïve PCa, with inconsistent findings (4, 5, 6). These discrepancies probably reflect differences in study design and methodology, including tissue sampling, pre-analytical processing, and analytical techniques for steroid quantification (7), underscoring the need for standardized approaches. Furthermore, the only study that directly quantified intraprostatic estradiol reported higher levels in PCa compared with benign prostatic hyperplasia (BPH) (8).

This hormonal dependency makes the prostate a plausible target for the adverse effects of endocrine-disrupting chemicals (EDCs). EDCs comprise a diverse group of natural and synthetic compounds capable of interfering with hormonal signaling and have been implicated in reproductive disorders, thyroid dysfunction, metabolic disease, obesity, neuroendocrine alterations, and hormone-dependent cancers (9, 10, 11).

Although several studies and reviews have addressed the potential role of EDCs in PCa development and progression (9, 12, 13), available human data remain limited and inconclusive. Associations between urinary or circulating EDC concentrations and PCa incidence or severity have been reported (11, 14), yet direct evidence linking intraprostatic EDCs to prostate carcinogenesis is lacking. Notably, no studies have directly compared circulating and intraprostatic EDC levels within the same individuals to determine whether serum measurements accurately reflect tissue burden. In our preliminary work, we demonstrated detectable concentrations of EDCs in cancerous prostate tissue (15), underscoring the biological plausibility of local retention.

The purpose of this study was to comprehensively characterize intraprostatic steroid and EDC profiles within the tumor microenvironment, adjacent non-cancerous tissues from peripheral and transition zones, and plasma in patients with localized PCa that underwent robot-assisted radical prostatectomy. Furthermore, we evaluated the association between intratumoral steroid and EDC levels and tumor aggressiveness, as defined by ISUP grade group classification, as well as prognosis, assessed by prostate-specific antigen (PSA) levels six months after surgery, and risk categories. Finally, we explored the potential integration of steroid hormones and EDCs into machine learning models to enhance patient risk classification.

## Methods

### Study sample and study design

This prospective observational cohort included 178 consecutive patients with histologically confirmed localized PCa undergoing robot-assisted radical prostatectomy with 6-month follow-up (see section on Supplementary materials given at the end of the article). For each patient, three tissue specimens were collected intraoperatively: one tumor sample, one histologically non-malignant sample from the peripheral zone, and one histologically non-malignant sample from the transition zone of the prostate. Peripheral venous blood was collected at three predefined time points: on the day of surgery (baseline) and 3 and 6 months postoperatively. Three patients were excluded from the final analysis due to prior neoadjuvant hormonal therapy. Two additional patients were excluded due to alternative histopathological diagnoses (neuroendocrine carcinoma and intraductal carcinoma). All remaining patients had adenocarcinoma predominantly located in the peripheral zone of the prostate, with one case originating in the transition zone. In total, data from 173 patients were available for statistical analyses. Baseline patient characteristics and clinicopathological outcomes are presented in Tables 1 and 2. As part of the statistical analyses, patients were stratified into four risk categories according to postoperative pathological assessment, reflecting the estimated risk of disease progression (Table S1).

**Table 1:** Baseline demographic, clinical, and oncological characteristics of consecutive patients undergoing robot-assisted radical prostatectomy. Continuous variables are presented as median (interquartile range), and categorical variables as *n* (%). Percentages for categorical distributions are calculated using available case denominators.

Patient characteristics	Total cohort (*n* = 173)
Patient characteristics	
Age (years), median (IQR)	65.5 (59.5–69.2)
BMI (kg/m^2^), median (IQR)	27.7 (25.1–30.8)
Past medical history, *n* (%)	
Hypertension	108 (62.4)
Diabetes mellitus	27 (15.6)
Coronary artery disease	44 (25.4)
Current smoker	23 (13.3)
Previous abdominal surgery	78 (45.1)
Prostate cancer characteristics	
PSA (ng/mL), median (IQR)	6.30 (4.70–8.60)
PSA category (ng/mL), *n* (%)	
<10	146 (84.4)
10–20	23 (13.3)
>20	4 (2.3)
Clinical T stage, *n* (%)	
cT1	115 (66.5)
cT2	57 (32.9)
cT3	1 (0.6)
Biopsy ISUP grade group, *n* (%)	
GG 1 (Gleason ≤ 6)	77 (44.5)
GG 2 (Gleason 3 + 4)	62 (35.8)
GG 3 (Gleason 4 + 3)	17 (9.8)
GG 4 (Gleason 8)	15 (8.7)
GG 5 (Gleason 9–10)	2 (1.2)
Preoperative risk stratification (EAU), *n* (%)	
Low risk	56 (32.4)
Intermediate risk	66 (38.2)
High risk	51 (29.5)

BMI, body mass index; cT, clinical tumor stage; EAU, European Association of Urology; GG, grade group; ISUP, International Society of Urological Pathology; IQR, interquartile range; PSA, prostate-specific antigen.

**Table 2:** Clinicopathological outcomes after robot-assisted radical prostatectomy. Continuous variables are presented as median (interquartile range), and categorical variables as *n* (%). pN0/pN1 is reported as *n*/*N* (%), where *N* denotes the number of patients who underwent pelvic lymph node dissection. Percentages for categorical distributions were calculated using available case denominators. PSA persistence was defined as PSA ≥ 0.1 ng/mL at the respective time point.

Clinicopathological characteristics	Total cohort (*n* = 173)
Pathological stage	
Pathological stage T, *n* (%)	
pT2	130 (75.1)
pT2a	13 (7.5)
pT2b	2 (1.2)
pT2c	115 (66.5)
pT3	43 (24.9)
pT3a	22 (12.7)
pT3b	21 (12.1)
Lymph node dissection and nodal status	
PLND performed, *n* (%)	31 (17.9)
pNX (no PLND), *n* (%)	142 (82.1)
pN0 (negative nodes), *n*/*N* (%)	21/31 (67.7)
pN1 (positive nodes), *n* (%)	10/31 (32.3)
Pathological grade	
Pathological ISUP grade group, *n* (%)	
GG 1 (Gleason ≤ 6)	26 (15.0)
GG 2 (Gleason 3 + 4)	100 (57.8)
GG 3 (Gleason 4 + 3)	28 (16.2)
GG 4 (Gleason 8)	12 (6.9)
GG 5 (Gleason 9–10)	7 (4.0)
Adverse histopathological features	
Lymphovascular invasion, *n* (%)	2 (1.2)
Perineural invasion (PNI), *n* (%)	157 (90.8)
Cribriform architecture, *n* (%)	21 (12.1)
Surgical margins	
Negative (R0), *n* (%)	124 (71.7)
Positive (R1), *n* (%)	49 (28.3)
Early oncological PSA dynamics	
PSA at 3 months (*n* = 167)	
PSA (ng/mL), median (IQR)	0.006 (0.006–0.015)
PSA undetectable, *n* (%)	153 (91.6%)
PSA persistence, *n* (%)	14 (8.4%)
PSA at 6 months (*n* = 161)	
PSA (ng/mL), median (IQR)	0.006 (0.006–0.013)
PSA undetectable, *n* (%)	145 (90.1)
PSA persistence, *n* (%)	16 (9.9)

GG, grade group; ISUP, International Society of Urological Pathology; IQR, interquartile range; PLND, pelvic lymph node dissection; pN0, no regional lymph node metastasis; pN1, regional lymph node metastasis; pNX, lymph nodes not assessed (no PLND performed); pT, pathological tumor stage; PSA, prostate-specific antigen; R0, negative surgical margins; R1, positive surgical margins.

### LC–MS/MS analysis of steroid hormones and endocrine-disrupting chemicals (EDCs)

Steroid hormones and EDCs were quantified using validated and published liquid chromatography–tandem mass spectrometry (LC–MS/MS) methods (15, 16, 17). The methods enabled the detection of 30 steroids in their unconjugated form (3 estrogens, 15 androgens, and 12 C21 steroids), 17 steroid conjugates, and 16 EDCs (7 bisphenols, 5 parabens, 2 benzophenones, and 2 phytoestrogens) in both conjugated and unconjugated forms. However, some EDCs (bisphenols BPZ and BPAP, nonylphenol, and benzophenone-1) were not detected in the majority of plasma and prostate tissue samples. Detailed information on sample processing is provided in the Supplementary Material (Supplementary Methods). Lower limits of quantification (LLOQs) are provided in Tables S2 and S3.

### Data processing

All values below LLOQ were replaced by LLOQ/√2 according to Hornung *et al.* (18). Subsequently, power transformation was applied to achieve normal distribution of the data using Statgraphics Centurion XVIII software (USA).

In the same statistical software, differences in steroid hormone and EDC concentrations in plasma, cancerous tissue, and non-cancerous tissue were evaluated using analysis of variance (ANOVA). For comparisons across tissues and plasma, only patients with complete data for all analyzed matrices were included (*n* = 100). Although the overall cohort comprised 173 patients, not all matrices were available for each individual; restricting the analysis to complete cases ensured comparability across matrices and avoided missing data. Comparisons of steroid and EDC levels across groups stratified by Gleason score and ISUP grade were also performed using ANOVA. Post hoc multiple comparisons were conducted using Fisher’s least significant difference (LSD) test with the significance level set at *P* < 0.05. Spearman’s correlations between plasmatic and tissue EDCs were performed in Medcalc®, version 23.3.7 (Belgium).

### Multivariate analysis

Bidirectional orthogonal projections to latent structures (O2PLS) were used to assess associations between steroids and EDCs (in different matrices: cancerous tissue and plasma) and risk-stratified groups as well as patient prognosis (PSA levels after 6 months) in the statistical software SIMCA-P v.12.0 (Umetrics AB, Sweden). For each OPLS model, all potential predictors (steroids, EDCs, BMI, and age) were included in the first step. Using variable importance statistics, irrelevant predictors were removed, and an improved model including only the relevant predictors was fitted. The F-distributed Hotelling’s T^2^ statistic and a normal probability plot were used to identify non-homogeneities, which were subsequently removed. Finally, the ‘distance to the model’ statistic was applied to identify values that were significantly distant from the model (*P* < 0.01); these were then eliminated. This procedure was repeated until all non-homogeneities and irrelevant variables were removed.

### Machine learning models

Data preprocessing for machine learning models consisted of the following steps:

- removal of outliers using the interquartile range (IQR) method with a factor of 1.5, removing samples with outliers in more than five features (the resulting sample counts per set are shown in Fig. 4, panel E (column N Samp));
- log-transformation (log1p) of steroid features exhibiting absolute skewness above 1.5;
- removal of highly correlated feature pairs (Pearson |*r*| > 0.8);
- stratified train/test split (75%/25%);
- feature selection to the top 15 features (analytes) based on ensemble importance ranking from four models (random forest, XGBoost, elastic net, and decision tree), with clinical features force-included for the augmented feature sets;
- robust scaling (centering by median and scaling by IQR). Missing values were imputed using median imputation. The data were split into 75% training and 25% test sets using stratified sampling. Model performance was evaluated using five-fold repeated stratified cross-validation with 10 repeats on the training set (50 total fold evaluations). In each CV fold, 80% of training data were used for model fitting (60% of total data) and 20% for validation (15% of total data). Accuracy, macro-averaged F1 score, and 95% bootstrap confidence intervals (1,000 iterations) were computed on the held-out test set. Early stopping with a patience of 200 iterations was applied using a 20% monitoring split from the fold training data.

The pipeline was implemented in Python using the CatBoost library (Yandex, Netherlands) for gradient boosting classification; scikit-learn for preprocessing, cross-validation, and evaluation; XGBoost and random forest for ensemble-based feature importance ranking; and pandas, Matplotlib, and seaborn for data handling and visualization, with code co-developed using Claude (Anthropic, USA).

A CatBoost machine learning classification model was used to distinguish risk categories using five different feature set configurations:

- preoperative clinical variables (age, BMI, cT stage, ISUP grade group (GG ISUP), and baseline PSA (PSA_0_); SET_A),
- preoperative clinical variables augmented with steroids and EDCs (SET_A_STEROIDS),
- postoperative clinical variables (age, BMI, pathological stage T (pT), pathological stage N (pN), pathological ISUP grade group (pGG ISUP), and baseline PSA (PSA_0_); SET_B),
- postoperative clinical variables augmented with steroids and EDCs (SET_B_STEROIDS), and
- steroids and EDCs only (ONLY_STEROIDS).

## Results

### Comparison of steroid and EDC levels in plasma (collected on the day of surgery), cancerous tissue, and non-cancerous tissues

For approximate comparison between plasma and tissue concentrations, a tissue density of 1 g/mL was assumed, and concentrations expressed in ng/g were treated as comparable to ng/mL. This simplification does not account for differences in tissue composition (e.g. lipid and water content), which may affect direct comparability. Plasma levels were compared with cancerous and non-cancerous prostate tissue (peripheral and transition zones) only for analytes with the majority of values above the LLOQ (Table 3), as differing LLOQs between matrices could otherwise produce artificial statistical significance.

**Table 3:** Medians and interquartile ranges (IQRs) of steroids and endocrine-disrupting chemicals (EDCs) in non-cancerous transition (ZT) and peripheral (ZP) zone tissues, tumor tissue (T), and plasma. Differences between the tumor sample and at least one non-tumor sample are highlighted in bold.

Analyte	ZT (ng/g; *n* = 128)	ZP (ng/g; *n* = 129)	T (ng/g; *n* = 100)	Plasma (ng/mL; *n* = 171)	Multiple comparisons
Part A (Steroid hormones)					
**Preg**	**1.326 (0.766; 2.436)**	**1.576 (0.871; 2.277)**	**1.712 (0.839; 3.087)**	**0.582 (0.371; 0.863)**	**T > ZT, P; ZP > P**
**PregC**	**791.2 (616.7; 1,084.3)**	**757.1 (563.4; 984.4)**	**745.0 (603.8; 912.5)**	**28.5 (18.5; 45.0)**	**ZT > T, P; ZP > P**
17OHPreg	1.241 (0.377; 2.76)	1.655 (0.673; 2.667)	1.546 (0.564; 3.471)	0.52 (0.283; 0.902)	ZT, ZP, T > P
17OHPregC	23.91 (18.26; 35.22)	24.70 (18.81; 33.40)	24.61 (18.07; 31.08)	n.d.	n.d.
DHEA	2.573 (1.174; 6.141)	2.839 (0.796; 5.843)	2.355 (0.377; 6.751)	1.672 (1.026; 2.771)	ZP > P
**DHEAC**	**109.8 (37.18; 186.9)**	**106.8 (40.5; 172.3)**	**79.4 (24.6; 140.6)**	**473.4 (308.5; 687.0)**	**P > ZP, ZT > T**
7α-HydroxyDHEA	0.151 (0.151; 0.263)	0.151 (0.151; 0.29)	0.151 (0.151; 0.311)	0.193 (0.127; 0.296)	T, ZP > P
7-ketoDHEA	0.084 (0.019; 0.166)	0.086 (0.019; 0.146)	0.097 (0.019; 0.16)	0.048 (0.022; 0.069)	ZT, ZP, T > P
7-ketoDHEAC	0.591 (0.372; 0.802)	0.539 (0.308; 0.794)	0.516 (0.346; 0.791)	0.133 (0.059; 0.229)	ZT, ZP, T > P
7β-HydroxyDHEA	0.189 (0.189; 0.189)	0.189 (0.189; 0.189)	0.189 (0.189; 0.189)	0.063 (0.041; 0.097)	ZT, ZP, T > P
17OHProg	0.075 (0.075; 0.075)	0.075 (0.075; 0.075)	0.075 (0.075; 0.075)	0.617 (0.401; 0.873)	P > ZT, ZP, T
17OHProgC	11.63 (8.67; 16.43)	11.17 (7.88; 16.15)	12.64 (8.90; 16.34)	0.93 (0.484; 1.418)	ZT, ZP, T > P
A4	0.019 (0.019; 0.028)	0.019 (0.019; 0.035)	0.019 (0.019; 0.038)	0.665 (0.498; 0.898)	P > ZT, ZP, T
**T**	**0.025 (0.009; 0.042)**	**0.025 (0.013; 0.053)**	**0.033 (0.018; 0.057)**	**3.385 (2.512; 4.456)**	**P > T > ZT, ZP**
TC	0.116 (0.057; 0.236)	0.144 (0.066; 0.242)	0.133 (0.067; 0.226)	0.631 (0.37; 0.979)	P > ZT, ZP, T
11KT	0.075 (0.075; 0.075)	0.075 (0.075; 0.075)	0.075 (0.075; 0.075)	0.194 (0.15; 0.268)	P > ZT, ZP, T
11OHT	0.038 (0.038; 0.038)	0.038 (0.038; 0.038)	0.038 (0.038; 0.038)	0.19 (0.142; 0.282)	P > ZT, ZP, T
**DHT**	**0.682 (0.409; 1.144)**	**0.831 (0.525; 1.291)**	**0.81 (0.544; 1.29)**	**0.209 (0.142; 0.279)**	**T, ZP > ZT > P**
DHTC	0.799 (0.574; 1.134)	0.833 (0.595; 1.133)	0.866 (0.602; 1.183)	n.d.	n.d.
E1	0.028 (0.028; 0.037)	0.028 (0.028; 0.043)	0.028 (0.028; 0.048)	0.026 (0.021; 0.033)	ZT, ZP, T > P
E1C	1.52 (1.235; 1.995)	1.398 (1.054; 1.908)	1.485 (1.131; 1.948)	0.403 (0.26; 0.585)	ZT, ZP, T > P
E2	0.032 (0.032; 0.032)	0.032 (0.032; 0.032)	0.032 (0.032; 0.032)	0.014 (0.01; 0.019)	n.d.
E2C	0.12 (0.071; 0.256)	0.189 (0.1; 0.43)	0.16 (0.095; 0.394)	0.022 (0.014; 0.035)	ZT, ZP, T > P
E3	0.028 (0.028; 0.028)	0.028 (0.028; 0.028)	0.028 (0.028; 0.028)	0.003 (0.003; 0.003)	n.d.
**E3C**	**2.182 (1.852; 2.842)**	**2.198 (1.795; 2.666)**	**2.09 (1.865; 2.675)**	**0.282 (0.218; 0.333)**	**ZT > ZP, T > P**
DHP	0.033 (0.019; 0.091)	0.033 (0.019; 0.091)	0.039 (0.019; 0.096)	0.014 (0.014; 0.014)	ZT, ZP, T > P
F	4.04 (2.15; 10.08)	4.45 (2.42; 9.06)	4.84 (3.01; 13.4)	130.68 (98.19; 165.26)	P > ZT, ZP, T
**E**	**1.32 (0.5; 3.93)**	**1.86 (0.77; 4.90)**	**2.84 (1.31; 8.76)**	**18.20 (15.29; 23.50)**	**P > T > ZP > ZT**
**B**	**0.069 (0.057; 0.166)**	**0.057 (0.057; 0.218)**	**0.1 (0.057; 0.277)**	**3.109 (1.719; 5.26)**	**P > T > ZP, ZT**
BC	0.282 (0.064; 4.443)	0.328 (0.146; 4.14)	0.424 (0.153; 4.394)	0.486 (0.166; 2.302)	P > ZT
**21DOF**	**0.028 (0.019; 0.053)**	**0.019 (0.019; 0.044)**	**0.019 (0.019; 0.043)**	**0.014 (0.008; 0.024)**	**ZT > ZP, T > P**
21DOFC	7.881 (5.32; 13.62)	8.11 (4.91; 11.89)	8.87 (5.82; 12.41)	0.72 (0.53; 0.99)	ZT, ZP, T > P


Analyte	ZT (ng/g; *n* = 128)	ZP (ng/g; *n* = 129)	T (ng/g; *n* = 100)	Plasma (ng/mL; *n* = 171)	Multiple comparisons
Part B (Endocrine disrupting chemicals)					
MP	0.48 (0.151; 2.405)	0.421 (0.151; 1.711)	0.541 (0.167; 2.142)	0.011 (0.011; 0.02)	ZT, ZP, T > P
**MPC**	**68.48 (27.77; 186.19)**	**40.65 (22.42; 76.46)**	**34.15 (18.43; 76.66)**	**6.89 (5.53; 9.05)**	**ZT > ZP, T > P**
EP	0.069 (0.028; 0.297)	0.05 (0.028; 0.226)	0.047 (0.028; 0.275)	0.003 (0.003; 0.003)	ZT, ZP, T > P
**EPC**	**0.818 (0.393; 2.397)**	**0.607 (0.342; 0.891)**	**0.489 (0.336; 0.997)**	**0.02 (0.012; 0.044)**	**ZT > ZP, T > P**
PP	0.684 (0.117; 3.812)	0.612 (0.202; 2.506)	0.736 (0.273; 2.68)	0.003 (0.003; 0.003)	ZT, ZP, T > P
**PPC**	**4.445 (2.587; 15.168)**	**3.989 (2.552; 6.793)**	**3.81 (2.193; 5.467)**	**0.025 (0.012; 0.059)**	**ZT > ZP, T > P**
BP	0.038 (0.038; 0.038)	0.038 (0.038; 0.038)	0.038 (0.038; 0.038)	0.003 (0.003; 0.003)	n.d.
BPC	0.086 (0.038; 0.239)	0.088 (0.038; 0.21)	0.111 (0.038; 0.27)	0.006 (0.003; 0.021)	ZT, ZP, T > P
BenzylP	0.028 (0.028; 0.047)	0.028 (0.028; 0.041)	0.028 (0.028; 0.028)	0.003 (0.003; 0.004)	n.d.
BPA	0.267 (0.151; 0.591)	0.276 (0.151; 0.619)	0.268 (0.151; 0.633)	0.011 (0.011; 0.029)	ZT, ZP, T > P
BPAC	1.699 (1.015; 2.779)	1.584 (0.83; 2.796)	1.775 (1.046; 2.759)	0.028 (0.011; 0.082)	ZT, ZP, T > P
BPS	0.333 (0.113; 0.738)	0.342 (0.139; 0.716)	0.314 (0.141; 0.761)	0.011 (0.011; 0.011)	ZT, ZP, T > P
BPSC	0.328 (0.131; 0.626)	0.307 (0.127; 0.606)	0.344 (0.132; 0.679)	0.011 (0.011; 0.011)	ZT, ZP, T > P
BPF	0.038 (0.038; 0.038)	0.038 (0.038; 0.038)	0.038 (0.038; 0.038)	0.003 (0.003; 0.003)	n.d.
BPFC	0.064 (0.038; 0.101)	0.063 (0.038; 0.103)	0.056 (0.038; 0.097)	0.007 (0.005; 0.01)	ZT, ZP, T > P
BPAFC	0.626 (0.489; 0.84)	0.63 (0.479; 0.812)	0.593 (0.481; 0.792)	0.022 (0.016; 0.032)	ZT, ZP, T > P
Oxybenzone	0.057 (0.057; 0.057)	0.057 (0.057; 0.093)	0.057 (0.057; 0.087)	0.01 (0.004; 0.017)	n.d.
OxybenzoneC	5.263 (2.687; 7.646)	5.605 (3.082; 8.92)	5.755 (3.312; 8.987)	0.346 (0.173; 0.473)	ZT, ZP, T > P
Daidzein	0.038 (0.038; 0.038)	0.038 (0.038; 0.038)	0.038 (0.038; 0.038)	0.003 (0.003; 0.003)	n.d.
DaidzeinC	0.038 (0.038; 0.107)	0.038 (0.038; 0.097)	0.038 (0.038; 0.137)	0.006 (0.003; 0.023)	n.d.
Genistein	0.038 (0.038; 0.038)	0.038 (0.038; 0.038)	0.038 (0.038; 0.038)	0.003 (0.003; 0.003)	n.d.
GenisteinC	0.077 (0.038; 0.242)	0.071 (0.038; 0.31)	0.057 (0.038; 0.224)	0.009 (0.003; 0.025)	ZT, ZP, T > P

C after the steroid hormone/EDC name indicates a conjugated form; Preg, pregnenolone; 17OHPreg, 17-hydroxypregnenolone; DHEA, dehydroepiandrosterone; 7α-hydroxyDHEA, 7α-hydroxydehydroepiandrosterone; 7-ketoDHEA, 7-ketodehydroepiandrosterone; 7β-hydroxyDHEA, 7β-hydroxydehydroepiandrosterone; 17OHProg, 17-hydroxyprogesterone; A4, androstenedione; T, testosterone; 11KT, 11-ketotestosterone; 11OHT, 11β-hydroxytestosterone; DHT, 5α-dihydrotestosterone; E1, estrone; E2, estradiol; E3, estriol; DHP, 5α-dihydroprogesterone; F, cortisol; E, cortisone; B, corticosterone; Aldo, aldosterone; 21DOF, 21-deoxycortisol; MP, methylparaben; EP, ethylparaben; PP, propylparaben; BP, butylparaben; benzylP, benzylparaben; BPA, bisphenol A; BPS, bisphenol S; BPF, bisphenol F; BPAF, bisphenol AF.

In all cases, EDC concentrations were significantly higher in cancerous and non-cancerous prostate tissues compared with plasma, suggesting tissue-specific bioaccumulation and retention.

**Figure 1:**
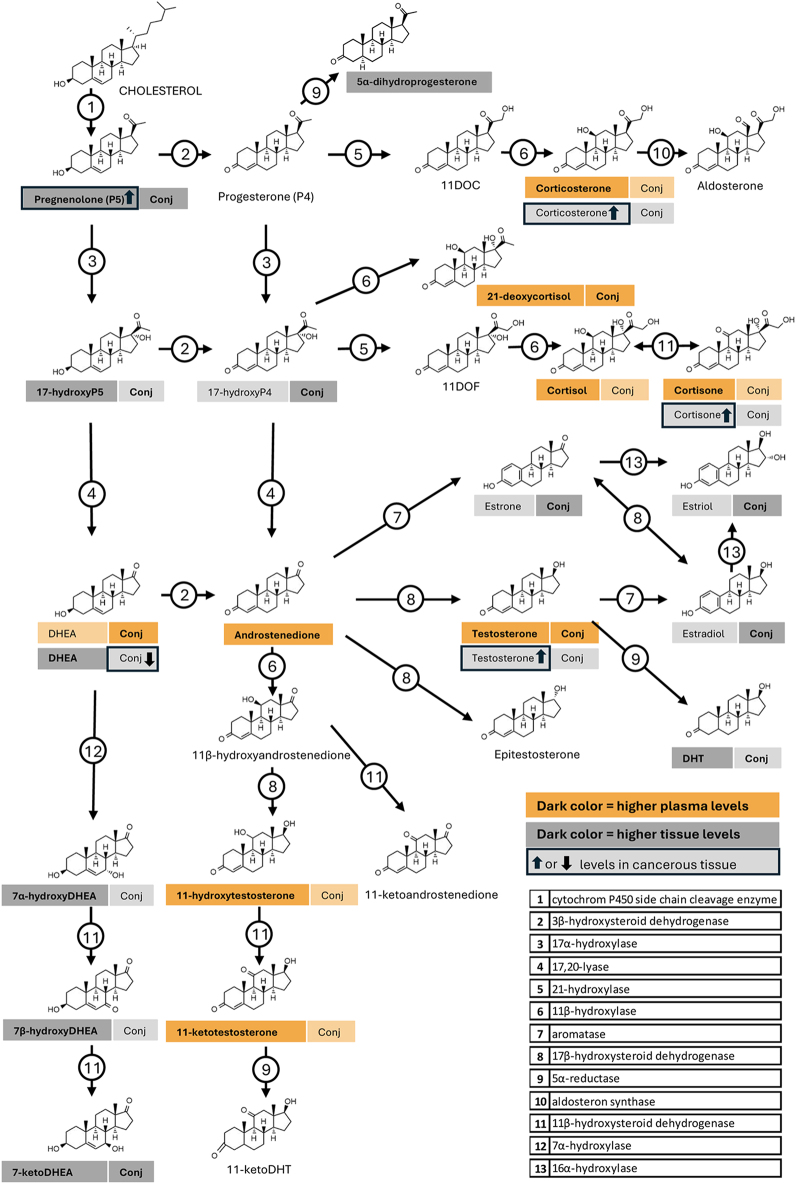
Steroid biosynthesis illustrating differences between prostate tissue and plasma and between cancerous and non-cancerous prostate tissues. A full color version of this figure is available at https://doi.org/10.1530/ERC-26-0153.

Compared with plasma, prostate tissue (both cancerous and non-cancerous) showed higher levels of pregnenolone and its conjugate, 17-hydroxypregnenolone, DHEA and its metabolites, 17-hydroxyprogesterone and its conjugate, DHT, conjugated estrogens, 5α-dihydroprogesterone, and 21-deoxycortisol and its conjugate. On the contrary, androgens (androstenedione, testosterone, and its derivatives) and corticoids (cortisol, cortisone, corticosterone and its conjugate) were higher in plasma (Fig. 1, Table 3).

**Figure 2:**
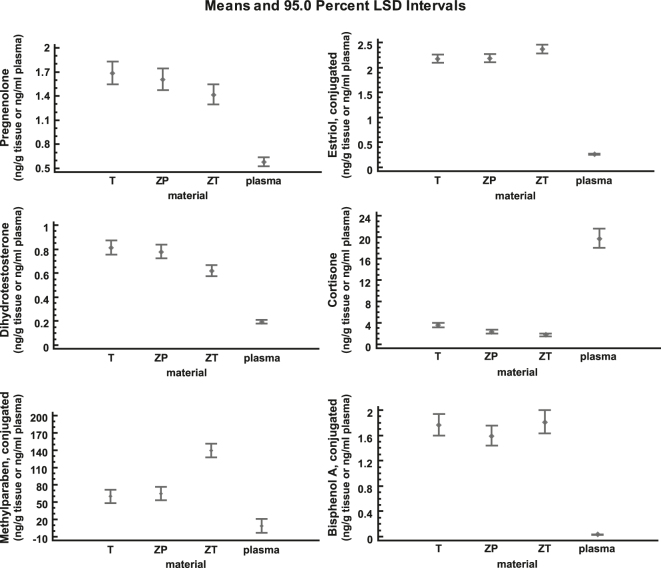
Comparison of selected analytes in cancerous tissue (tumor = T), non-cancerous tissue from the peripheral zone (ZP) and the transition zone (ZT), and plasma. Values are expressed as mean and 95% Fisher’s LSD interval.

Compared with non-cancerous tissues from both zones, tumors showed significantly lower conjugated DHEA (DHEAC) and higher testosterone, cortisone, and corticosterone levels. Additional significant differences are presented in Table 3 and Fig. 2.

### Correlations between plasma and tumor tissue levels of EDCs

Spearman correlations between plasma EDC concentrations and EDC levels in prostate tumor tissue are summarized in Table 4. In general, plasma EDC concentrations did not correlate with corresponding tissue levels. Exceptions were observed for phytoestrogens, with the strongest correlations identified for conjugated daidzein (*r* = 0.516, *P* < 0.001) and conjugated genistein (r = 0.442, *P* < 0.001) between plasma and tissue. In addition, a moderate correlation was observed between plasma conjugated MP and its tissue counterpart (*r* = 0.297, *P* = 0.003). The complete correlogram of plasma and prostate tissue EDC levels is presented in Table S4. Furthermore, the correlogram of intratumoral EDCs within prostate tissue is shown in Table S5, where particularly strong correlations were observed among parabens.

**Table 4:** Spearman correlations between plasma and intratumoral prostatic tissue endocrine-disrupting chemicals (EDCs). Statistically significant correlations are highlighted in bold.

EDC	*r*	*P*-value
MP	0.001	0.989
MPC	**0.297**	**0.003**
EP	−0.082	0.424
EPC	0.134	0.194
PP	−0.061	0.551
PPC	0.034	0.741
BPC	−0.124	0.229
BPA	0.030	0.773
BPAC	0.009	0.928
BPS	−0.057	0.580
BPSC	−0.113	0.275
BPF	0.015	0.887
BPFC	−0.005	0.958
BPAFC	−0.123	0.232
Oxybenzone	0.164	0.108
OxybenzoneC	−0.109	0.291
Daidzein	**0.239**	**0.018**
DaidzeinC	**0.516**	**<0.0001**
Genistein	0.113	0.271
GenisteinC	**0.442**	**<0.0001**

C after steroid name indicates a conjugated form; MP, methylparaben; EP, ethylparaben; PP, propylparaben; BP, butylparaben; benzylP, benzylparaben; BPA, bisphenol A; BPS, bisphenol S; BPF, bisphenol F; BPAF, bisphenol AF.

### Comparison of individual steroids and EDCs across pathological ISUP grade groups (and GS)

A comparison of individual steroids and EDCs according to pathological ISUP (pISUP) and pGS is presented in Tables S6 and S7. Higher-grade tumors showed elevated levels of 7ketoDHEAC, androstenedione, conjugated testosterone, 5α-dihydroprogesterone, cortisone, cortisol, estrone conjugate, butylparaben (conjugated and unconjugated), and oxybenzone. In contrast, estradiol conjugate was higher in pISUP 2 vs pISUP 5, and EPC was higher in pISUP 1 compared with pISUP 2 and pISUP 4. Among 11-oxygenated androgens, 11-ketotestosterone was higher in pGS 9 vs pGS 7, and 11β-hydroxytestosterone in pGS 6 vs pGS 7; however, these findings should be interpreted cautiously due to the majority of values being below the LLOQ.

### Associations of steroid hormones and EDCs with prognosis expressed as PSA level at 6 months using multivariate prediction models

**Figure 3:**
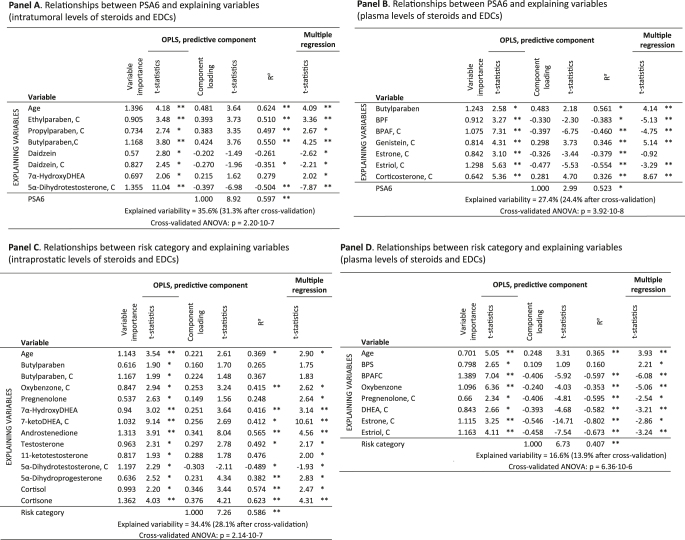
Relationships between explained variable and explaining variables as evaluated by models of orthogonal projections to latent structure (OPLS) and ordinary multiple regression (OMR). aR – component loadings expressed as correlation coefficients with predictive component, *P < 0.05, **P < 0.01; C after steroid/EDC name indicates a conjugated form.

Associations between steroids, EDCs, and patient prognosis were evaluated using O2PLS models based on intratumoral (Fig. 3, panel A) and plasma levels (Fig. 3, panel B), with age and BMI included as explaining variables. In the intratumoral model, reflecting the hormonal composition of the tumor microenvironment, age, conjugated parabens (ethyl-, propyl-, and butylparaben), and 7α-hydroxyDHEA were positively associated with PSA at 6 months, whereas conjugated DHT and daidzein (conjugated and unconjugated) were negatively associated, indicating a more favorable postoperative course.

The plasma-based model (Fig. 3, panel B), reflecting the systemic hormonal milieu, explained less PSA6 variability than the intratumoral model but revealed several significant associations. Higher plasma levels of butylparaben, conjugated genistein, and conjugated corticosterone were positively associated with PSA6, whereas BPF, conjugated BPAF, and conjugated estrogens were negatively associated. Thus, although plasma profiles captured less variability, systemic hormonal and EDC exposure remained linked to early postoperative PSA dynamics.

### Associations of steroids and EDCs with risk categories by multivariate prediction models

Associations between risk categories and steroids/EDCs were evaluated using two O2PLS models based on intratumoral and plasma levels. Age and BMI were included as explaining variables in both models. In both models, higher age was associated with higher-risk groups.

In cancerous tissue (Fig. 3, panel C), a higher steroid pool – driven by pregnenolone, corticoids, and androgens including 11-ketotestosterone – was associated with higher risk categories. In contrast, higher DHT levels were linked to lower risk. Among EDCs, conjugated oxybenzone was associated with higher risk, while butylparaben (conjugated and unconjugated) showed a similar but non-significant association but was included in the final model.

In plasma (Fig. 3, panel D), different relationships and predictors were observed; age and higher BPS levels were associated with higher risk, whereas BPAF, oxybenzone, and conjugated steroids (pregnenolone, DHEA, and estrogens) were associated with lower risk.

Overall, the findings indicate a dissociation between systemic and intratumoral metabolism: efficient systemic conjugation of steroids and EDCs was associated with lower risk, whereas their intratumoral accumulation and active steroidogenesis were linked to higher-risk disease. The intratumoral OPLS model explained substantially more variability (34.4%; 28.1% after cross-validation) than the plasma-based O2PLS model (16.6%; 13.9%), indicating that intratumoral profiles better captured variance related to risk stratification.

### Machine learning (ML)-based classification into risk categories

**Figure 4:**
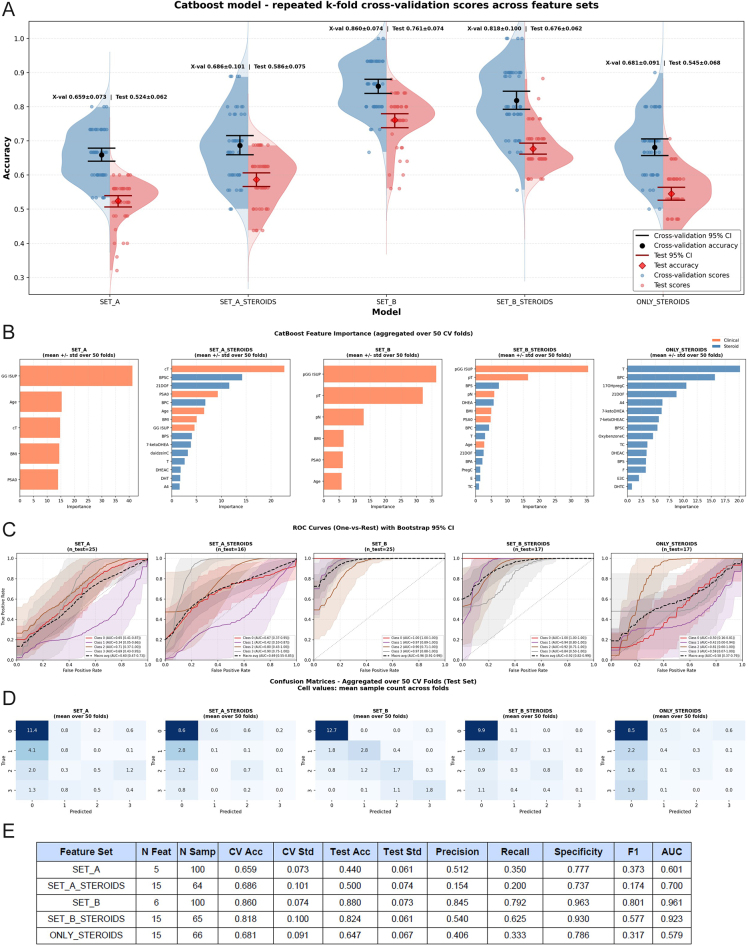
Comparison of model performance for each set in terms of cross-validation and test accuracy, visualized using violin plots (panel A), CatBoost feature importance (panel B), ROC curves (panel C), confusion matrix (panel D), and results for each feature set (panel E). Cross-validation accuracy (CV Acc) and cross-validation standard deviation (CV Std) are computed on the validation folds (held-out portion of the training data in each CV fold). All remaining metrics (test accuracy, precision, recall, specificity, F1, and AUC) are macro-averaged and computed on the held-out 25% test set, which was never seen during training or cross-validation. A full color version of this figure is available at https://doi.org/10.1530/ERC-26-0153.

Finally, a CatBoost model was applied to classify patients into risk categories using five datasets: presurgical data without and with intratumoral steroids/EDCs (SET_A and SET_A_STEROIDS), postsurgical data without and with steroids/EDCs (SET_B and SET_B_STEROIDS), and steroids/EDCs only (ONLY_STEROIDS) (Fig. 4). Postoperative models are shown as an upper performance bound, as the target variable is partially derived from input features. The clinically relevant comparison is between SET_A and SET_A_STEROIDS, reflecting presurgical decision-making. Inclusion of intratumoral steroids and EDCs improved classification accuracy and reduced overfitting compared with the clinical model alone (Fig. 4, panel E). The ONLY_STEROIDS model further supported their relevance, achieving 65% test accuracy (68% cross-validation (CV) accuracy).

Key variables included C21 steroids (conjugated 17-hydroxypregnenolone, 21-deoxycortisol, and cortisol), androgens (testosterone and DHT in both forms and androstenedione), DHEA-related metabolites, conjugated estriol, and the EDCs (conjugated butylparaben, oxybenzone, and bisphenol S in both forms) (Fig. 4, panel B). These findings align with the O2PLS model, which linked risk categories to corticoids, androgens, conjugated butylparaben, and oxybenzone.

## Discussion

In this study, we compared steroid and EDC profiles in cancerous and non-cancerous prostate tissue, alongside corresponding plasma levels, in patients with localized PCa. We demonstrated that intratumoral steroid and EDC signatures differ from systemic profiles and are associated with tumor aggressiveness, postoperative PSA dynamics, and patient risk stratification.

Prostate tissue consistently exhibited substantially higher concentrations of EDCs, especially the conjugated ones (sulfated), compared with circulating levels, indicating local bioaccumulation and retention within the gland. Furthermore, plasma and tissue EDC levels generally did not correlate, with some exceptions, particularly for phytoestrogens. Consequently, plasma measurements may not fully reflect the biologically relevant intratumoral EDC environment when assessing the potential role of EDCs in PCa pathogenesis. This may partly explain the inconsistent findings reported in previous studies, which predominantly relied on measurements in biological matrices other than the target tissue.

In humans, tissue concentrations of various EDCs have been most investigated in breast (tumor) tissue (19, 20, 21), adipose tissue (22), and placenta (23, 24). Parabens, particularly methylparaben (MP), have been detected in human breast tumor tissue at median levels of 16.6 ng/g (range: 0–5,102.9 ng/g) (20) and mean concentrations of 12.8 ± 2.2 ng/g (19) and 178.47 ± 107.10 ng/g (21). The latter study that also compared malignant and adjacent non-malignant breast tissue did not observe significant differences in paraben concentrations between these tissue types (21). Although malignant tissues exhibit altered lipid metabolism and composition compared with non-malignant tissues (as reviewed in (25, 26)), which could theoretically influence EDC retention, such effects did not appear to significantly affect paraben concentrations, either in the study by Amin *et al.* (21) or in the present study.

On the other hand, tissue-specific accumulation of parabens has been reported in small cetaceans, with markedly higher concentrations of methylparaben in the liver, kidney, and stomach compared with lipid-rich tissues (27). Similarly, paired adipose tissue and plasma samples from 14 adults showed no accumulation of MP in adipose tissue relative to serum (28), suggesting preferential distribution to non-adipose tissues, which is consistent with our findings in prostate tissue.

In our study, conjugated parabens (methyl-, ethyl-, and propylparaben) were detected in prostate tissue at higher concentrations in the transition zone than in the peripheral zone. This difference may reflect the greater stromal and fibromuscular composition of the transition zone versus the predominantly glandular peripheral zone. The higher paraben content observed in the transition zone may be of particular relevance in the context of BPH, which originates in this region.

Bisphenol A (BPA) has been detected in human adipose tissue in the low ng/g range (29, 30, 31), generally at higher concentrations than MP (29). This may reflect greater lipophilicity of BPA compared with MP. BPA has also been reported at higher concentrations in placenta than in maternal blood (32), although not consistently across studies (24). In our study, BPA levels were higher in prostate tissue than in plasma, but the tissue–plasma gradient was less pronounced than for parabens. This may relate to preferential partitioning into lipid-rich tissues (33) and extensive first-pass metabolism, as BPA is predominantly glucuronidated (34). In our analytical approach, we quantified primarily the sulfated fraction. In contrast, MP is metabolized via both glucuronidation and sulfation, with sulfated forms representing a substantial proportion of circulating metabolites (35).

Steroid distribution differed by class, with steroid precursors and several estrogen-related metabolites enriched in tissue, whereas circulating androgens and corticoids predominated in plasma, reflecting compartment-specific steroid metabolism. Importantly, cancerous tissue demonstrated a distinct steroid profile characterized by reduced DHEA conjugates and increased testosterone and corticoid levels compared with both types of non-cancerous tissue, suggesting tumor-specific alterations in local steroid metabolism.

Previous studies investigating intraprostatic androgen levels in PCa in comparison with BPH have reported inconsistent findings (4, 5, 6, 8). In addition to differences in study design and analytical methodologies, this variability may also reflect differences in patient populations, particularly in the distribution of GS/ISUP grades, which were not always considered in previous studies. In our cohort, differences in steroid levels according to the GS/ISUP grade were observed, particularly for androgens (androstenedione, conjugated testosterone, and 7ketoDHEAC), corticosteroids (cortisol and corticosterone), and 5α-dihydroprogesterone. Consistent with these findings, previous studies have reported lower DHT levels in more aggressive PCa compared with less aggressive disease (36, 37). In our cohort, a similar non-significant trend was observed for conjugated DHT. Together, these findings suggest that intraprostatic steroid metabolism may vary according to tumor aggressiveness, as reflected by grade-associated alterations across steroid pathways rather than isolated changes in individual steroids, and indicate that GS/ISUP stratification should be considered when interpreting steroid profiles in PCa tissue.

Enhanced levels of corticoids within the prostate tumor microenvironment represent a novel observation and were positively associated with tumor aggressiveness and clinical risk stratification. Consistent with these findings, recent evidence indicates dysregulation of glucocorticoid receptor expression in treatment-naïve tumors, characterized by either loss or overexpression (38), suggesting a potential role of cortisol-mediated signaling in tumor progression. Elevated intratumoral cortisol levels have also been reported in association with higher malignancy in endometrial cancer (39). Given the established immunomodulatory effects of glucocorticoids (40), local corticoid activity may contribute to tumor immune evasion (41). Together, these findings suggest that altered intratumoral glucocorticoid metabolism may contribute to the biological behavior of aggressive PCa and represent a previously underappreciated component of the prostate tumor microenvironment. If confirmed in independent cohorts, intratumoral corticoid profiles may also have potential as biomarkers of tumor aggressiveness or as targets for modulation of glucocorticoid signaling pathways.

Higher DHT levels in prostate compared with circulation were reported previously (42) and were confirmed in our study. Our finding that higher intratumoral DHT levels were associated with low-risk disease and improved prognosis may appear counterintuitive, given the established role of DHT in prostatic growth. However, DHT primarily drives physiological and benign prostatic growth (43), as illustrated by the absence of prostate development in 5α-reductase deficiency (e.g. Imperato-McGinley syndrome). This suggests that DHT-dependent growth reflects a regulated, differentiation-associated state of prostatic epithelium. During malignant transformation, steroidogenesis may shift toward a more dysregulated intratumoral steroid milieu that promotes tumor progression, immune evasion, and systemic signaling. Consistent with this concept, patients with more aggressive disease have been reported to exhibit lower intraprostatic DHT levels (36, 37). In this context, reduced DHT-driven signaling and expansion of alternative steroid pathways may reflect metabolic reprogramming toward a more aggressive phenotype.

In benign prostate, 5α-reductase type 2 (SRD5A2) predominates, whereas PCa progression involves reduced SRD5A2 and upregulated type 1 (SRD5A1), although the mechanisms of this switch remain unclear (44). Despite increased 5α-reductase expression, lower intraprostatic DHT levels in aggressive tumors, as observed in our cohort (non-significant trend) and previously reported (36, 37), may reflect altered substrate utilization. Preferential conversion of alternative substrates, such as progesterone, may divert steroid metabolism from DHT. Consistently, aggressive tumors in our cohort exhibited elevated 5α-dihydroprogesterone with undetectable progesterone, supporting increased enzymatic conversion (45), suggesting that progesterone metabolism may represent an alternative sink for 5α-reductase activity in aggressive PCa.

The observed alterations in intratumoral steroid profiles, both relative to non-cancerous tissue and across different ISUP grade groups, may be associated with pathways involved in the development of castration-resistant PCa. Importantly, signatures of these changes were detectable at the time of diagnosis when the tumor was still organ-confined, suggesting their potential relevance for early risk stratification and refinement of personalized therapeutic strategies.

A major strength of this study is its prospective, consecutively enrolled, and rigorously characterized patient cohort with comprehensive clinical, clinicopathological, and biological data collected at predefined time points. The relatively short follow-up period represents a limitation of this study, as prognosis was inferred from PSA levels measured 6 months after surgery. Although early PSA dynamics after radical prostatectomy are established predictors of long-term outcomes, including biochemical recurrence, metastasis-free survival, and cancer-specific mortality, this limited follow-up precludes direct assessment of these endpoints. Another limitation is the lack of an independent external validation cohort. However, the OPLS models were internally validated using seven-fold cross-validation (venetian blinds, SIMCA), with Q^2^ and p(CV-ANOVA) values reported in the results tables. The machine learning (CatBoost) models were validated using repeated stratified five-fold cross-validation and a strictly held-out test set excluded from all stages of model development. A further limitation of the multivariate and machine learning models is the imbalance between risk groups, largely reflecting the prospective study design and the predominance of intermediate-risk patients in the cohort. Future validation in an independent cohort will be important to confirm the generalizability of the present findings.

## Conclusion

This is the first comprehensive evaluation of intraprostatic steroid metabolism and EDCs in both prostate tissue and plasma in PCa. We demonstrated that tumor steroidogenesis shifts toward an expanded intratumoral steroid pool, characterized by elevated corticoids and increased testosterone and estrone conjugates, with reduced DHEA sulfate compared with non-cancerous tissue. All detected EDCs were present at higher concentrations in prostate tissue than in plasma, and tissue and plasma levels were generally poorly correlated, with the exception of phytoestrogens. EDC exposure was associated with adverse prognosis (conjugated parabens), tumor aggressiveness (butylparaben and oxybenzone), and higher-risk categories in multivariable and machine learning models. Importantly, integration of intratumoral steroid and EDC profiles into a presurgical clinical model improved risk stratification, underscoring the diagnostic potential of steroidomic and EDC profiling in PCa.

## Supplementary materials



## Declaration of interest

The authors declare that there is no conflict of interest that could be perceived as prejudicing the impartiality of the work reported.

## Funding

This work was supported by the Ministry of Health of the Czech Republic in cooperation with the Czech Health Research Council under project No. NU21J-01-00040 and MH CZ – DRO, Institute of Endocrinology EÚ, 00023761.

## Author contribution statement

JV contributed to writing of the original draft, conceptualization, supervision, and funding acquisition. TS contributed to review and editing, validation, and methodology (LC–MS/MS analysis). AV contributed to writing of the original draft and methodology (LC–MS/MS analysis). LG and MH contributed to review and editing and methodology (clinical examinations and surgeries). MN contributed to review and editing and methodology (histopathological examinations). MH contributed to review and editing and methodology (statistical analyses). MS contributed to writing of the original draft, conceptualization, and supervision. LK contributed to review and editing and methodology (data evaluation and artwork). AL contributed to review and editing and methodology (deconjugation of steroids and EDCs). JH contributed to writing of the original draft, conceptualization, supervision, and project administration.

## AI disclosure

Artificial intelligence tools (ChatGPT 5.3, OpenAI) were used solely for language editing, grammar correction, and improving text flow.

## Ethics statement

This study was carried out according to the principles of the Declaration of Helsinki (World Medical Association, 2000). The study protocol was approved by the Ethics Committee of the Institute of Endocrinology and the Military University Hospital Prague. Written informed consent for the collection and research use of biological materials was obtained from all participants prior to enrollment.
