# Supplementary material for: Intraprostatic steroid hormones and endocrine disruptors in prostate cancer
Source: Endocr Relat Cancer. 2026 Jul 7;33(7):e260153. doi: 10.1530/ERC-26-0153 (PMC13386167; doi:10.1530/ERC-26-0153)
Supplement: Supplementary file 1 [file ERC-26-0153_supplementary_materials.pdf]

Supplementary material

Intraprostatic steroid hormones and endocrine disruptors in prostate cancer

Jana Vitku[1*], Tereza Skodova[1], Anezka Varausova[1], Lukas Gadus[2,3], Michal Horenitzky[2,3], Marie Novakova[4], Martin Hill[1], Michaela Svojtkova[1], Lucie Kolatorova[1], Adela Lukaskova[1], Jiri Heracek[2,3]

[1]Department of Steroids and Proteofactors, Institute of Endocrinology, Prague, Czech Republic
[2] Department of Urology, First Faculty of Medicine, Charles University, Prague, Czech Republic
[3] Department of Urology, Military University Hospital, Prague, Czech Republic
[4] Department of Pathology, Military University Hospital, Prague, Czech Republic

*Corresponding author: Jana Vitku (maiden name: Kubatova)
Email address: jvitku@endo.cz

Table of Contents

Supplementary methods 2

1. Surgical procedure 2

2. Histopathological evaluation 2

3. LC-MS/MS analysis of steroid hormones and endocrine-disrupting chemicals (EDCs) 2

3.1 Chemicals and reagents 2

3.2 Preparation of plasma samples for the analysis of unconjugated steroid hormones and EDCs 3

3.3 Preparation of tissue samples for the analysis of unconjugated steroid hormones and EDCs .. 3

3.4 Preparation of samples for the analysis of conjugated steroids and EDCs from both matrices 3

3.5 Liquid chromatography-tandem mass spectrometry (LC-MS/MS) 4

Table S1. Postoperative risk stratification schema for patients undergoing robot-assisted radical prostatectomy 5

Table S2 Lower limits of quantification (LLOQ) for steroid hormones in plasma and prostate tissue 6

Table S3. Lower limits of quantification (LLOQ) for endocrine-disrupting chemicals (EDCs) in plasma and prostate tissue 7

Table S4. Spearman correlations between plasma and intratumoral prostate tissue EDC levels 8

Table S5. Spearman correlations between EDC levels within intratumoral prostate tissue 9

Table S6. Medians and interquartile ranges (IQR) of steroid hormones in cancerous tissue by pathological ISUP grade (pISUP), with multiple comparisons across post-surgical ISUP grades and Gleason scores (pGS). 10

Table S7. Medians and interquartile ranges (IQR) of EDCs in cancerous tissue by post-surgical ISUP grade (pISUP), with multiple comparisons across post-surgical ISUP grades and Gleason scores (pGS). 11

References 12

Supplementary methods

1. Surgical procedure

Robotic-assisted radical prostatectomy were performed using the da Vinci Xi robotic system (Intuitive Surgical, Sunnyvale, CA, USA). An extraperitoneal or transperitoneal approach was selected based on anatomical considerations and the planned extent of lymph node dissection. The surgery was performed in a 25 ° Trendelenburg position using a standardized port configuration. After bladder-neck dissection, posterior dissection with control of the vascular pedicles, and complete mobilization of the seminal vesicles, unilateral or bilateral nerve preservation was performed in selected patients based on preoperative risk stratification. The dorsal venous complex was ligated and the urethra transected at the apex; A urethrovesical anastomosis was fashioned with a continuous single-layer suture. Pelvic lymph node dissection, which encompasses the external iliac, obturator, and internal iliac territories, was performed selectively in patients at intermediate or high risk of nodal involvement according to the EAU Guidelines.

2. Histopathological evaluation

The prostate sample obtained during surgery was topographically marked by the operating surgeon and immediately transported to the Department of Pathology. Three tissue samples were collected by an experienced pathologist according to prostatic topography: one from the macroscopic tumor lesion and two from macroscopically tumor-free tissue (one from the peripheral zone and one from the transition zone). All tissue specimens analysed in the study comprised intraprostatic parenchymal tissue (either tumour tissue or histologically non-malignant prostatic tissue from the peripheral or transition zone); periprostatic adipose tissue was not sampled.

Each specimen, approximately 10 mm in diameter, was divided into two halves. Half was processed according to standard histopathological protocols and served for histological verification of the analyzed tissue. The other half was immediately placed into cryotubes using metal forceps and designated for steroid and EDC analyses. Samples were stored at −80 °C until analysis.

3. LC-MS/MS analysis of steroid hormones and endocrine-disrupting chemicals (EDCs)

3.1 Chemicals and reagents

The chemicals and materials used for the chromatography and sample preparation were obtained from the following suppliers: steroids, EDCs and internal standards were purchased from Cambridge Isotope Laboratories, Inc. (Tewksbury, MA, USA), Steraloids (Newport, RI, USA), Merck (Darmstadt, Germany), Toronto Research Chemicals Inc. (Toronto, ON, Canada), Koch-Light Laboratories Ltd. (Suffolk, UK), Chromservis (Prague, Czech Republic), Chiron (Trondheim, Norway), EQ Laboratories GmbH (Augsburg, Germany), Cayman Chemical Company (Ann Arbor, MI, USA), and Chiron (Trondheim, Norway). Methanol (≥99.9%) and water were obtained from Honeywell Research Chemicals (Charlotte, NC, USA). Ethyl acetate (≥99.9%) and n-hexane (≥99%) were purchased from VWR International (Wayne, PA, USA). Formic acid (≥96%) and ammonium fluoride (≥98.0%) were supplied by VWR International (Stribrna Skalice, Czech Republic. A 0.9% saline solution (0.9% sodium chloride) was supplied by Ardeapharma, a.s. (Sevetin, Czech Republic). All solvents and reagents were of LCMS grade.

3.2 Preparation of plasma samples for the analysis of unconjugated steroid hormones and EDCs

A plasma sample (500 µL) was transferred into a glass tube, spiked with 10 µL of the internal standard (IS) mixture, and diluted with 500 µL of 0.9% saline, followed by vortexing. Liquid–liquid extraction was performed using 2 mL of hexane : ethyl acetate (v/v, 3:2) with mixing for 1 min. The organic phase was then transferred to a clean glass tube and evaporated to dryness at 25 °C using a vacuum concentrator. The residues were reconstituted in 100 µL of 50% methanol and centrifuged at 4650 × g for 5 min. The clarified supernatants were transferred to LC vials with glass inserts. All details of the validated method can be found in previous publications [1,2].

3.3 Preparation of tissue samples for the analysis of unconjugated steroid hormones and EDCs

A frozen prostate tissue sample (approximately 30 mg) was wrapped in aluminum foil, immersed in liquid nitrogen, and mechanically pulverized using a hammer. The homogenized tissue was transferred into a glass tube and accurately weighed. Subsequently, 1 mL of 0.9% saline was added, and liquid–liquid extraction was performed using hexane: ethyl acetate (v/v, 3:2; 3 mL; 2mins). The aqueous phase was frozen using dry ice, enabling the organic phase to be decanted into a clean glass tube. The organic extract was evaporated to dryness in a vacuum concentrator. The residues were reconstituted in 100 µL of 50% methanol, and centrifuged at 4650 × g for 5 min. The resulting supernatants were transferred into vials with glass inserts for LC–MS/MS analysis.

More details can be found in Vitku et al 2023 [3] where a fully validated method for the analysis of estrogens and EDCs in negative ESI mode is described. The same sample preparation procedure was used for all analytes, including EDCs, estrogens, and other steroids. Androgens and C21 steroids were subsequently analyzed in positive ESI mode. The LC–MS/MS method for androgens and C21 steroids has been fully validated for plasma[2]. For prostate tissue samples, partial validation was performed to confirm its applicability to this matrix. This partial validation included an assessment of within-run accuracy and precision at three concentration levels in pooled prostate homogenates, each analyzed in triplicate (20 mg of tissue per sample). All evaluated steroid concentrations met acceptance criteria, remaining within ±15% of nominal values. Coefficients of variation were within ±20% at the LLOQ level and within ±15% at medium and high concentration levels. Potential matrix effects were minimized through the use of deuterated internal standards for each analyte. In addition, potential interferences were evaluated using structurally related compounds available in our laboratory (including anabolic steroids and androstane and pregnane isomers), and no significant interferences were observed.

3.4 Preparation of samples for the analysis of conjugated steroids and EDCs from both matrices

The aqueous phase containing residual conjugated steroids and EDCs was precipitated by adding 2 mL of ice-cold methanol. The samples were centrifuged at 3000 rpm for 15 min, and the resulting supernatants were transferred into clean glass tubes and evaporated to dryness under reduced pressure. Subsequently, chemical deconjugation was performed using trimethylchlorosilane (TMCS). Briefly, 500 µL of 1 M TMCS was added to each dried sample, followed by incubation at 55 °C for 1 h. After deconjugation, approximately 100 mg of sodium bicarbonate was added to neutralize the reaction mixture, and the samples were again evaporated to dryness. The final residues were reconstituted in 100 µL of 50% methanol and processed in the same manner as unconjugated steroids. All procedures were conducted under controlled conditions to minimize potential contamination EDs, in accordance with previously published protocols [4].

3.5 Liquid chromatography-tandem mass spectrometry (LC-MS/MS)

Each analytical batch comprised two calibration sets, study samples, and quality control (QC) samples and procedural blanks to control contamination. The LC-MS/MS measurements were performed with an Exion LC AD system connected to a Sciex QTRAP 6500 + mass spectrometer (Sciex, Concord, USA) using two LC-MS/MS methods; one in positive ESI mode, second in ESI negative mode. For the measurements in positive ESI mode Kinetex C18 column (100 mm × 3.0 mm, 2.6 µm) and Security Guard ULTRA cartridge system (UHPLC C18 for the 3 mm ID column) were used. For negative mode Kinetex Biphenyl column (100 mm × 3 mm, 2.6 µm) with the corresponding Security Guard ULTRA cartridge system (UHPL C18 for the 3 mm ID Biphenyl column) were used, all purchased from Phenomenex (Torrance, CA, USA). A sample volume of 20 µL for the positive mode and 30 µL for the negative mode was injected into the LC-MS/MS system for analysis.

The mobile phase solvents for the positive mode were 0.1 % formic acid (FA) in water (eluent A) and 0.1 % FA in methanol (eluent B). Water and methanol were used as mobile phases in negative mode, with 6 mM ammonium fluoride (NH_4F) in the water used for post-column infusion. More information about methods and instrument setting can be found at [1,5]. Following the implementation of updated ICH M10 on bioanalytical method validation guideline [6], the lower limits of quantification (LLOQ) for individual steroid hormones and endocrine disruptors were recalculated and are reported in Tables S2 and S3, respectively.

Table S1. Postoperative risk stratification schema for patients undergoing robot-assisted radical prostatectomy. Risk assignment is determined by a combination of pathological nodal status (pN), pathological tumor stage (pT), prostate-specific antigen level at 3 months after surgery, ISUP grade group on the definitive prostatectomy specimen, and surgical margin status. Each patient was classified into the highest applicable risk group. Pathological staging was performed according to the EAU Guidelines. PSA persistence was defined as PSA ≥ 0.1 ng/mL at 3 months after radical prostatectomy. The surgical margin classification distinguishes four categories based on the extent and number of positive margin foci.

Risk group	pN	pT	PSA (ng/mL) 3 mo post-RP	ISUP GG (specimen)	Surgical margins
Low	pN0/pNX	pT2	< 0.1	1–2	any
Intermediate	pN0/pNX	pT2	< 0.1	3	any
Intermediate	pN0/pNX	pT3a	< 0.1	1–3	R0 / R01 / focal R1
High	pN0/pNX	pT2	< 0.1	4–5	any
High	pN0/pNX	pT3a	< 0.1	4–5	R0 / R01 / focal R1
High	pN0/pNX	pT3a	< 0.1	any	extensive R1
High	pN0/pNX	pT3b–pT4	< 0.1	any	any
Very high	pN0/pNX	any	≥ 0.1	any	any
Very high	pN1/pNX	any	any	any	any

Surgical margin classification

Category	Definition
R0	Negative surgical margins (tumor-free resection margin)
R01	Margin ≤ 1 mm, single focus
Focal R1	Margin > 1 and ≤ 3 mm, single focus
Extensive R1	Margin > 3 mm, or ≥ 2 foci

Abbreviations: EAU, European Association of Urology; GG, grade group; ISUP, International Society of Urological Pathology; mo, months; pN, pathological nodal status; pN0, no regional lymph node metastasis; pN1, regional lymph node metastasis; pNX, regional lymph nodes not assessed; PSA, prostate-specific antigen; pT, pathological tumor stage; R0, negative surgical margins; R01, close margin (≤ 1 mm); R1, positive surgical margins; RP, radical prostatectomy.

Table S2 Lower limits of quantification (LLOQ) for steroid hormones in plasma and prostate tissue

Steroid	Abbreviation	LLOQ plasma (ng/mL)	LLOQ prostate tissue (ng/g)
Pregnenolone	Preg	0.020	0.267
17-Hydroxypregnenolone	17OHPreg	0.080	0.533
Dehydroepiandrosterone	DHEA	0.080	0.533
7α-Hydroxydehydroepiandrosterone	7α-HydroxyDHEA	0.020	0.213
7-ketodehydroepiandrosterone	7-ketoDHEA	0.010	0.027
7β-Hydroxydehydroepiandrosterone	7β-HydroxyDHEA	0.020	0.267
17-Hydroxyprogesterone	17OHProg	0.020	0.107
Androstenedione	A4	0.020	0.027
11-ketoandrostenedione	11KA4	0.020	n.d.
11β-Hydroxyandrostenedione	11bOHA4	0.080	n.d.
Testosterone	T	0.040	0.013
11-ketotestosterone	11KT	0.010	0.107
11β-Hydroxytestosterone	11OHT	0.004	0.053
Epitestosterone	EpiT	0.004	0.000
5α-Dihydrotestosterone	DHT	0.010	0.053
Estrone	E1	0.004	0.040
Estradiol	E2	0.004	0.045
Estriol	E3	0.004	0.040
5α-Dihydroprogesterone	DHP	0.020	0.027
Cortisol	F	1.600	0.267
Cortisone	E	0.240	0.133
Corticosterone	B	0.160	0.080
Aldosterone	Aldo	0.004	n.d.
21-Deoxycortisol	21DOF	0.002	0.027
11-Deoxycortisol	11DOF	0.020	n.d.
11-Deoxycorticosterone	11DOC	0.010	n.d.

n.d., not determined; 11DOC, 11DOF, 11βOHA4, 11KA4, and Aldo were not detectable in prostate tissue.

The LLOQ was defined as the lowest calibration standard with a concentration within ±20% of the nominal value. Additionally, for tissue samples, measured concentrations were normalized to tissue weight using the mean tissue mass per tube (37.5 mg) and expressed as ng/g tissue.

Table S3. Lower limits of quantification (LLOQ) for endocrine-disrupting chemicals (EDCs) in plasma and prostate tissue

EDC	Abbreviation	LLOQ plasma (ng/mL)	LLOQ prostate tissue (ng/g)
Methylparaben	MP	0.016	0.213
Ethylparaben	EP	0.004	0.040
Propylparaben	PP	0.004	0.053
Butylparaben	BP	0.004	0.053
Benzylparaben	benzylP	0.004	0.040
Bisphenol A	BPA	0.016	0.213
Bisphenol S	BPS	0.016	0.107
Bisphenol F	BPF	0.004	0.053
Bisphenol AF	BPAF	0.008	0.400
Bisphenol AP	BPAP	0.004	0.053
Bisphenol Z	BPZ	0.004	0.053
Bisphenol P	BPP	0.004	0.027
4-n-Nonylphenol	NP	0.004	0.053
Benzophenon-1		0.004	n.d.
Oxybenzone		0.004	0.080
Daidzein		0.004	0.053
Genistein		0.004	0.053

n.d., not determined; the standard for benzophenone-1 was not available at the time of prostate tissue sample analysis.
The LLOQ was defined as the lowest calibration standard with a concentration within ±20% of the nominal value. Additionally, for tissue samples, measured concentrations were normalized to tissue weight using the mean tissue mass per tube (37.5 mg) and expressed as ng/g tissue.

Table S4. Spearman correlations between plasma and intratumoral prostate tissue EDC levels

	**Intratumoral prostate tissue** endocrine disrupting chemicals																			
**Plasma** endocrine disrupting chemicals	**MP**	**MPC**	**EP**	**EPC**	**PP**	**PPC**	**BPC**	**BPA**	**BPAC**	**BPS**	**BPSC**	**BPF**	**BPFC**	**BPAFC**	**oxyben-zone**	**oxyben-zoneC**	**daid-zein**	**daid-zeinC**	**geni-stein**	**geni-steinC**
**MP**	0.001<br>*p=0.989*	0.076<br>*p=0.461*	0.052<br>*p=0.612*	0.012<br>*p=0.907*	-0.011<br>*p=0.912*	0.04<br>*p=0.700*	0<br>*p=0.998*	0.037<br>*p=0.721*	-0.015<br>*p=0.881*	0.092<br>*p=0.368*	-0.001<br>*p=0.992*	-0.02<br>*p=0.843*	-0.05<br>*p=0.628*	-0.106<br>*p=0.302*	0.171<br>*p=0.094*	-0.002<br>*p=0.987*	-0.023<br>*p=0.822*	0.051<br>*p=0.621*	-0.017<br>*p=0.869*	0.121<br>*p=0.239*
**MPC**	**0.292**<br>***p=0.004***	**0.297**<br>***p=0.003***	0.173<br>*p=0.091*	**0.291**<br>***p=0.004***	**0.244**<br>***p=0.016***	**0.277**<br>***p=0.006***	-0.007<br>*p=0.945*	-0.076<br>*p=0.457*	0.072<br>*p=0.484*	-0.109<br>*p=0.289*	0.045<br>*p=0.666*	-0.036<br>*p=0.728*	-0.096<br>*p=0.352*	-0.02<br>*p=0.846*	0.05<br>*p=0.624*	-0.104<br>*p=0.312*	0.166<br>*p=0.104*	0.127<br>*p=0.216*	-0.008<br>*p=0.940*	0.04<br>*p=0.702*
**EP**	-0.03<br>*p=0.769*	0.017<br>*p=0.869*	-0.082<br>*p=0.424*	0.046<br>*p=0.655*	0<br>*p=0.998*	-0.033<br>*p=0.751*	-0.026<br>*p=0.800*	-0.027<br>*p=0.796*	0.115<br>*p=0.267*	0.074<br>*p=0.468*	-0.08<br>*p=0.437*	-0.077<br>*p=0.454*	0.054<br>*p=0.599*	-0.118<br>*p=0.254*	0.13<br>*p=0.205*	0.031<br>*p=0.764*	0.078<br>*p=0.445*	0.19<br>*p=0.064*	0.008<br>*p=0.934*	0.122<br>*p=0.235*
**EPC**	-0.052<br>*p=0.615*	-0.042<br>*p=0.683*	-0.115<br>*p=0.262*	0.134<br>*p=0.194*	-0.066<br>*p=0.521*	-0.03<br>*p=0.775*	-0.05<br>*p=0.631*	0.004<br>*p=0.967*	**0.226**<br>***p=0.027***	0.073<br>*p=0.478*	0.144<br>*p=0.162*	**0.226**<br>***p=0.026***	-0.047<br>*p=0.649*	-0.031<br>*p=0.761*	0.136<br>*p=0.184*	0.014<br>*p=0.895*	-0.087<br>*p=0.399*	0.065<br>*p=0.532*	-0.162<br>*p=0.113*	0.086<br>*p=0.405*
**PP**	-0.076<br>*p=0.461*	0.123<br>*p=0.232*	0.123<br>*p=0.228*	0.115<br>*p=0.264*	-0.061<br>*p=0.551*	0.037<br>*p=0.718*	-0.04<br>*p=0.700*	0.088<br>*p=0.392*	0.047<br>*p=0.647*	0.14<br>*p=0.171*	0.123<br>*p=0.232*	0.035<br>*p=0.730*	0.063<br>*p=0.544*	-0.103<br>*p=0.317*	0.159<br>*p=0.120*	-0.017<br>*p=0.868*	0.02<br>*p=0.843*	0.13<br>*p=0.206*	-0.075<br>*p=0.468*	0.122<br>*p=0.236*
**PPC**	0.068<br>*p=0.505*	0.031<br>*p=0.762*	0.058<br>*p=0.575*	0.076<br>*p=0.460*	0.079<br>*p=0.442*	0.034<br>*p=0.741*	-0.028<br>*p=0.788*	-0.026<br>*p=0.802*	0.144<br>*p=0.162*	-0.039<br>*p=0.707*	0.04<br>*p=0.700*	0.053<br>*p=0.608*	-0.019<br>*p=0.854*	-0.163<br>*p=0.113*	0.122<br>*p=0.232*	-0.138<br>*p=0.180*	-0.089<br>*p=0.386*	0.094<br>*p=0.361*	-0.022<br>*p=0.830*	-0.077<br>*p=0.457*
**BPC**	0.052<br>*p=0.616*	-0.067<br>*p=0.520*	-0.014<br>*p=0.895*	0.031<br>*p=0.766*	0.034<br>*p=0.744*	-0.015<br>*p=0.882*	-0.124<br>*p=0.229*	0.046<br>*p=0.656*	**0.208**<br>***p=0.042***	-0.003<br>*p=0.976*	0.035<br>*p=0.733*	0.173<br>*p=0.090*	0.089<br>*p=0.388*	-0.09<br>*p=0.384*	0.067<br>*p=0.515*	-0.139<br>*p=0.178*	-0.126<br>*p=0.218*	-0.046<br>*p=0.657*	-0.119<br>*p=0.247*	-0.042<br>*p=0.686*
**BPA**	0.008<br>*p=0.936*	0.009<br>*p=0.930*	0.071<br>*p=0.491*	0.026<br>*p=0.804*	-0.024<br>*p=0.817*	-0.038<br>*p=0.715*	0.063<br>*p=0.545*	0.03<br>*p=0.773*	0.022<br>*p=0.832*	-0.069<br>*p=0.505*	0.005<br>*p=0.965*	-0.173<br>*p=0.090*	0.192<br>*p=0.062*	-0.079<br>*p=0.442*	-0.024<br>*p=0.818*	-0.074<br>*p=0.472*	0.057<br>*p=0.578*	-0.039<br>*p=0.705*	0.02<br>*p=0.842*	-0.023<br>*p=0.827*
**BPAC**	0.073<br>*p=0.477*	-0.025<br>*p=0.812*	-0.047<br>*p=0.651*	-0.079<br>*p=0.445*	0.029<br>*p=0.779*	-0.124<br>*p=0.228*	-0.135<br>*p=0.190*	-0.084<br>*p=0.415*	0.009<br>*p=0.928*	0.009<br>*p=0.932*	**-0.218**<br>***p=0.033***	-0.106<br>*p=0.300*	0.087<br>*p=0.401*	0.093<br>*p=0.369*	0.061<br>*p=0.554*	0.068<br>*p=0.510*	0.034<br>*p=0.741*	-0.004<br>*p=0.971*	-0.174<br>*p=0.088*	0.016<br>*p=0.881*
**BPS**	0.107<br>*p=0.297*	0.087<br>*p=0.402*	-0.038<br>*p=0.714*	-0.044<br>*p=0.673*	0.103<br>*p=0.313*	0.06<br>*p=0.560*	0.167<br>*p=0.104*	-0.172<br>*p=0.091*	-0.045<br>*p=0.661*	-0.057<br>*p=0.580*	0.015<br>*p=0.885*	-0.013<br>*p=0.900*	-0.075<br>*p=0.468*	0.151<br>*p=0.141*	-0.074<br>*p=0.474*	0.171<br>*p=0.095*	0.009<br>*p=0.928*	0.175<br>*p=0.088*	-0.007<br>*p=0.948*	0.013<br>*p=0.901*
**BPSC**	0.024<br>*p=0.816*	0.123<br>*p=0.233*	-0.004<br>*p=0.968*	0.092<br>*p=0.374*	0.001<br>*p=0.991*	0.089<br>*p=0.391*	-0.004<br>*p=0.967*	0.003<br>*p=0.974*	0.143<br>*p=0.165*	-0.176<br>*p=0.085*	-0.113<br>*p=0.275*	0.128<br>*p=0.212*	0.079<br>*p=0.442*	-0.002<br>*p=0.986*	-0.008<br>*p=0.934*	-0.023<br>*p=0.824*	0.032<br>*p=0.752*	0.099<br>*p=0.339*	0.003<br>*p=0.978*	0.12<br>*p=0.243*
**BPF**	-0.048<br>*p=0.639*	-0.053<br>*p=0.608*	-0.14<br>*p=0.171*	0.017<br>*p=0.873*	-0.081<br>*p=0.428*	0.023<br>*p=0.822*	-0.015<br>*p=0.887*	-0.017<br>*p=0.868*	-0.086<br>*p=0.407*	-0.062<br>*p=0.547*	-0.058<br>*p=0.578*	0.015<br>*p=0.887*	-0.001<br>*p=0.996*	0.058<br>*p=0.578*	**-0.218**<br>***p=0.032***	-0.072<br>*p=0.487*	0.112<br>*p=0.274*	0.075<br>*p=0.470*	-0.152<br>*p=0.138*	0.103<br>*p=0.318*
**BPFC**	0.107<br>*p=0.296*	-0.044<br>*p=0.672*	0.156<br>*p=0.127*	0.006<br>*p=0.957*	0.099<br>*p=0.335*	0.129<br>*p=0.210*	0.178<br>*p=0.082*	**0.207**<br>***p=0.042***	0.185<br>*p=0.071*	-0.089<br>*p=0.387*	0.157<br>*p=0.128*	0.09<br>*p=0.382*	-0.005<br>*p=0.958*	**0.229**<br>***p=0.025***	0.064<br>*p=0.537*	**0.212**<br>***p=0.038***	0.047<br>*p=0.646*	-0.081<br>*p=0.430*	**0.2**<br>***p=0.050***	-0.025<br>*p=0.808*
**BPAFC**	**0.211**<br>***p=0.038***	0.137<br>*p=0.182*	0.097<br>*p=0.344*	0.107<br>*p=0.301*	0.147<br>*p=0.151*	0.054<br>*p=0.598*	**-0.214**<br>***p=0.037***	0.053<br>*p=0.604*	-0.046<br>*p=0.657*	0.119<br>*p=0.245*	-0.106<br>*p=0.304*	-0.137<br>*p=0.180*	0.147<br>*p=0.154*	-0.123<br>*p=0.232*	0.123<br>*p=0.230*	0.013<br>*p=0.898*	0.176<br>*p=0.085*	0.008<br>*p=0.941*	0.049<br>*p=0.630*	0.049<br>*p=0.636*
**oxy-benzone**	-0.022<br>*p=0.834*	-0.026<br>*p=0.801*	0.045<br>*p=0.662*	-0.03<br>*p=0.771*	-0.056<br>*p=0.585*	-0.034<br>*p=0.742*	0.092<br>*p=0.374*	0.172<br>*p=0.092*	**0.303**<br>***p=0.003***	0.028<br>*p=0.786*	0.076<br>*p=0.463*	0.007<br>*p=0.944*	0.129<br>*p=0.211*	**0.331**<br>***p=0.001***	0.164<br>*p=0.108*	**0.276**<br>***p=0.007***	0.163<br>*p=0.112*	0.011<br>*p=0.915*	0.043<br>*p=0.678*	**0.219**<br>***p=0.032***
**oxy-benzoneC**	0.189<br>*p=0.064*	0.13<br>*p=0.208*	0.12<br>*p=0.241*	0.158<br>*p=0.124*	0.159<br>*p=0.120*	0.139<br>*p=0.176*	0.002<br>*p=0.981*	-0.127<br>*p=0.214*	0.006<br>*p=0.950*	-0.084<br>*p=0.412*	-0.015<br>*p=0.882*	-0.059<br>*p=0.569*	0.113<br>*p=0.274*	-0.031<br>*p=0.766*	-0.055<br>*p=0.594*	-0.109<br>*p=0.291*	0.004<br>*p=0.970*	0.09<br>*p=0.381*	0.129<br>*p=0.206*	0.097<br>*p=0.347*
**daidzein**	0.019<br>*p=0.857*	0.152<br>*p=0.138*	-0.008<br>*p=0.938*	**0.228**<br>***p=0.025***	0.038<br>*p=0.710*	**0.217**<br>***p=0.034***	**0.332**<br>***p=0.001***	0.076<br>*p=0.458*	-0.03<br>*p=0.769*	0.013<br>*p=0.900*	0.105<br>*p=0.311*	0.134<br>*p=0.190*	0.027<br>*p=0.796*	-0.053<br>*p=0.609*	-0.04<br>*p=0.697*	0.051<br>*p=0.622*	**0.239**<br>***p=0.018***	**0.289**<br>***p=0.004***	**0.295**<br>***p=0.003***	**0.25**<br>***p=0.014***
**daidzeinC**	0.13<br>*p=0.205*	**0.227**<br>***p=0.026***	0.089<br>*p=0.385*	0.2<br>*p=0.051*	0.118<br>*p=0.250*	0.189<br>*p=0.065*	0.092<br>*p=0.371*	-0.095<br>*p=0.357*	0.01<br>*p=0.922*	0.108<br>*p=0.292*	0.127<br>*p=0.218*	0.04<br>*p=0.699*	0.003<br>*p=0.975*	0.149<br>*p=0.148*	-0.043<br>*p=0.672*	0.198<br>*p=0.053*	**0.323**<br>***p=0.001***	**0.516**<br>***p<0.0001***	0.117<br>*p=0.254*	**0.474**<br>***p<0.0001***
**genistein**	0.021<br>*p=0.840*	0.089<br>*p=0.389*	0.054<br>*p=0.599*	**0.261**<br>***p=0.010***	0.013<br>*p=0.902*	0.076<br>*p=0.461*	**0.237**<br>***p=0.020***	-0.06<br>*p=0.561*	-0.012<br>*p=0.906*	0.098<br>*p=0.339*	0.117<br>*p=0.257*	-0.083<br>*p=0.417*	-0.082<br>*p=0.427*	**0.229**<br>***p=0.025***	-0.097<br>*p=0.344*	**0.204**<br>***p=0.046***	**0.206**<br>***p=0.043***	**0.28**<br>***p=0.006***	0.113<br>*p=0.271*	**0.317**<br>***p=0.002***
**genisteinC**	0.192<br>*p=0.060*	0.177<br>*p=0.084*	**0.214**<br>***p=0.035***	**0.206**<br>***p=0.044***	0.179<br>*p=0.079*	**0.25**<br>***p=0.014***	**0.256**<br>***p=0.012***	-0.168<br>*p=0.101*	0.128<br>*p=0.213*	0.018<br>*p=0.859*	0.157<br>*p=0.127*	-0.031<br>*p=0.765*	0.097<br>*p=0.350*	0.06<br>*p=0.559*	-0.004<br>*p=0.973*	**0.274**<br>***p=0.007***	0.082<br>*p=0.427*	**0.292**<br>***p=0.004***	-0.041<br>*p=0.693*	**0.442**<br>***p<0.0001***

Table S5. Spearman correlations between EDC levels within intratumoral prostate tissue

	PPC	MPC	PP	MP	EP	EPC	BPSC	benzylP	BPA	BPAC	genisteinC	BPS	daidzeinC	oxyben-zoneC	BP	daidzein	genistein	BPAFC	BPF	BPC	SequolC	BPFC	oxyben-zone
**PPC**	1	**0.698**<br>***p<0.0001***	**0.617**<br>***p<0.0001***	**0.626**<br>***p<0.0001***	**0.611**<br>***p<0.0001***	**0.747**<br>***p<0.0001***	**0.319**<br>***p=0.0016***	0.153<br>*p=0.137*	0.148<br>*p=0.151*	**0.253**<br>***p=0.013***	**0.201**<br>***p=0.0508***	0.066<br>*p=0.525*	0.167<br>*p=0.103*	0.147<br>*p=0.152*	0.114<br>*p=0.270*	0.122<br>*p=0.238*	0.194<br>*p=0.059*	0.119<br>*p=0.250*	**0.218**<br>***p=0.033***	**0.265**<br>***p=0.009***	-0.075<br>*p=0.465*	0.055<br>*p=0.592*	0.014<br>*p=0.894*
**MPC**	**0.698**<br>***p<0.0001***	1	**0.588**<br>***p<0.0001***	**0.566**<br>***p<0.0001***	**0.65**<br>***p<0.0001***	**0.69**<br>***p<0.0001***	**0.29**<br>***p=0.0042***	0.069<br>*p=0.506*	**0.258**<br>***p=0.011***	0.037<br>*p=0.720*	**0.237**<br>***p=0.020***	0.181<br>*p=0.078*	0.165<br>*p=0.109*	0.105<br>*p=0.308*	0.042<br>*p=0.688*	**0.205**<br>***p=0.045***	0.098<br>*p=0.343*	0.022<br>*p=0.834*	0.117<br>*p=0.256*	0.082<br>*p=0.428*	**0.279**<br>***p=0.006***	0.048<br>*p=0.644*	0.117<br>*p=0.258*
**PP**	**0.617**<br>***p<0.0001***	**0.588**<br>***p<0.0001***	1	**0.933**<br>***p<0.0001***	**0.702**<br>***p<0.0001***	**0.439**<br>***p<0.0001***	0.131<br>*p=0.203*	0.052<br>*p=0.610*	0.091<br>*p=0.376*	0.107<br>*p=0.301*	0.095<br>*p=0.355*	0.136<br>*p=0.185*	0.026<br>*p=0.802*	0.032<br>*p=0.759*	0.036<br>*p=0.724*	0.148<br>*p=0.149*	**0.233**<br>***p=0.022***	0.032<br>*p=0.760*	0.038<br>*p=0.714*	0.133<br>*p=0.196*	0.086<br>*p=0.403*	-0.064<br>*p=0.536*	0.047<br>*p=0.648*
**MP**	**0.626**<br>***p<0.0001***	**0.566**<br>***p<0.0001***	**0.933**<br>***p<0.0001***	1	**0.679**<br>***p<0.0001***	**0.408**<br>***p<0.0001***	0.175<br>*p=0.088*	0.032<br>*p=0.758*	0.12<br>*p=0.242*	0.131<br>*p=0.205*	0.056<br>*p=0.586*	0.124<br>*p=0.226*	0.036<br>*p=0.727*	0.084<br>*p=0.416*	0.003<br>*p=0.975*	**0.195**<br>***p=0.055***	**0.25**<br>***p=0.014***	0.134<br>*p=0.195*	0.006<br>*p=0.954*	0.097<br>*p=0.348*	0.098<br>*p=0.342*	-0.015<br>*p=0.886*	0.067<br>*p=0.517*
**EP**	**0.611**<br>***p<0.0001***	**0.65**<br>***p<0.0001***	**0.702**<br>***p<0.0001***	**0.679**<br>***p<0.0001***	1	**0.532**<br>***p<0.0001***	0.171<br>*p=0.095*	0.093<br>*p=0.363*	0.077<br>*p=0.451*	0.09<br>*p=0.383*	0.2<br>*p=0.051*	0.019<br>*p=0.851*	0.124<br>*p=0.230*	0.145<br>*p=0.159*	0.11<br>*p=0.284*	**0.221**<br>***p=0.030***	**0.226**<br>***p=0.026***	0.011<br>*p=0.917*	0.003<br>*p=0.979*	0.123<br>*p=0.234*	0.158<br>*p=0.123*	0.096<br>*p=0.350*	0.006<br>*p=0.953*
**EPC**	**0.747**<br>***p<0.0001***	**0.69**<br>***p<0.0001***	**0.439**<br>***p<0.0001***	**0.408**<br>***p<0.0001***	**0.532**<br>***p<0.0001***	1	0.181<br>*p=0.078*	-0.044<br>*p=0.674*	-0.046<br>*p=0.657*	0.072<br>*p=0.484*	**0.232**<br>***p=0.023***	0.022<br>*p=0.830*	0.12<br>*p=0.246*	0.111<br>*p=0.282*	-0.119<br>*p=0.249*	0.133<br>*p=0.198*	0.115<br>*p=0.264*	0.017<br>*p=0.872*	0.09<br>*p=0.384*	0.182<br>*p=0.076*	0.032<br>*p=0.756*	0.049<br>*p=0.636*	-0.024<br>*p=0.814*
**BPSC**	**0.319**<br>***p=0.002***	**0.29**<br>***p=0.004***	0.131<br>*p=0.203*	0.175<br>*p=0.088*	0.171<br>*p=0.095*	0.181<br>*p=0.078*	1	**0.348**<br>***p=0.0005***	**0.472**<br>***p<0.0001***	**0.442**<br>***p<0.0001***	**0.282**<br>***p=0.005***	**0.525**<br>***p<0.0001***	**0.29**<br>***p=0.004***	0.097<br>*p=0.346*	**0.252**<br>***p=0.013***	0.118<br>*p=0.254*	0.009<br>*p=0.928*	0.085<br>*p=0.410*	**0.308**<br>***p=0.002***	0.134<br>*p=0.193*	0.086<br>*p=0.404*	0.166<br>*p=0.106*	0.072<br>*p=0.488*
**benzylP**	0.153<br>*p=0.137*	0.069<br>*p=0.506*	0.052<br>*p=0.610*	0.032<br>*p=0.758*	0.093<br>*p=0.363*	-0.044<br>*p=0.674*	**0.348**<br>***p=0.0005***	1	**0.479**<br>***p<0.0001***	**0.364**<br>***p=0.0003***	**0.267**<br>***p=0.009***	**0.291**<br>***p=0.004***	0.129<br>*p=0.210*	0.129<br>*p=0.212*	**0.536**<br>***p<0.0001***	0.104<br>*p=0.312*	**0.235**<br>***p=0.021***	**0.234**<br>***p=0.022***	**0.363**<br>***p=0.0003***	-0.09<br>*p=0.383*	-0.012<br>*p=0.909*	**0.346**<br>***p=0.001***	0.15<br>*p=0.144*
**BPA**	0.148<br>*p=0.151*	**0.258**<br>***p=0.011***	0.091<br>*p=0.376*	0.12<br>*p=0.242*	0.077<br>*p=0.451*	-0.046<br>*p=0.657*	**0.472**<br>***p<0.0001***	**0.479**<br>***p<0.0001***	1	**0.373**<br>***p=0.0002***	0.072<br>*p=0.485*	**0.522**<br>***p<0.0001***	-0.063<br>*p=0.544*	0.039<br>*p=0.709*	**0.365**<br>***p=0.0002***	0.046<br>*p=0.655*	0.032<br>*p=0.758*	0.184<br>*p=0.073*	**0.279**<br>***p=0.006***	-0.18<br>*p=0.080*	0.166<br>*p=0.107*	0.103<br>*p=0.316*	**0.341**<br>***p=0.001***
**BPAC**	**0.253**<br>***p=0.013***	0.037<br>*p=0.720*	0.107<br>*p=0.301*	0.131<br>*p=0.205*	0.09<br>*p=0.383*	0.072<br>*p=0.484*	**0.442**<br>***p<0.0001***	**0.364**<br>***p=0.0003***	**0.373**<br>***p=0.0002***	1	0.195<br>*p=0.057*	**0.228**<br>***p=0.026***	0.098<br>*p=0.341*	**0.295**<br>***p=0.004***	**0.34**<br>***p=0.0007***	0.004<br>*p=0.972*	0.028<br>*p=0.790*	**0.268**<br>***p=0.008***	**0.242**<br>***p=0.017***	**0.226**<br>***p=0.027***	-0.123<br>*p=0.231*	0.144<br>*p=0.162*	**0.265**<br>***p=0.009***
**genisteinC**	**0.201**<br>***p=0.050***	**0.237**<br>***p=0.020***	0.095<br>*p=0.355*	0.056<br>*p=0.586*	0.2<br>*p=0.051*	**0.232**<br>***p=0.023***	**0.282**<br>***p=0.0053***	**0.267**<br>***p=0.009***	0.072<br>*p=0.485*	**0.195**<br>***p=0.057***	1	**0.277**<br>***p=0.006***	**0.585**<br>***p<0.0001***	0.13<br>*p=0.206*	-0.012<br>*p=0.906*	**0.333**<br>***p=0.001***	0.148<br>*p=0.149*	0.057<br>*p=0.582*	0.125<br>*p=0.223*	0.079<br>*p=0.442*	0.186<br>*p=0.069*	**0.247**<br>***p=0.015***	0.026<br>*p=0.800*
**BPS**	0.066<br>*p=0.525*	0.181<br>*p=0.078*	0.136<br>*p=0.185*	0.124<br>*p=0.226*	0.019<br>*p=0.851*	0.022<br>*p=0.830*	**0.525**<br>***p<0.0001***	**0.291**<br>***p=0.004***	**0.522**<br>***p<0.0001***	**0.228**<br>***p=0.027***	**0.277**<br>***p=0.006***	1	0.192<br>*p=0.061*	-0.088<br>*p=0.396*	0.115<br>*p=0.260*	0.048<br>*p=0.637*	-0.112<br>*p=0.273*	0.025<br>*p=0.811*	**0.233**<br>***p=0.021***	**-0.21**<br>***p=0.040***	**0.287**<br>***p=0.005***	0.169<br>*p=0.100*	**0.218**<br>***p=0.032***
**daidzeinC**	0.167<br>*p=0.103*	0.165<br>*p=0.109*	0.026<br>*p=0.802*	0.036<br>*p=0.727*	0.124<br>*p=0.230*	0.12<br>*p=0.246*	**0.29**<br>***p=0.004***	0.129<br>*p=0.210*	-0.063<br>*p=0.544*	0.098<br>*p=0.341*	**0.585**<br>***p<0.0001***	0.192<br>*p=0.061*	1	0.024<br>*p=0.814*	0.102<br>*p=0.322*	**0.44**<br>***p<0.0001***	**0.279**<br>***p=0.006***	-0.061<br>*p=0.552*	0.052<br>*p=0.617*	0.046<br>*p=0.653*	**0.337**<br>***p=0.0008***	0.04<br>*p=0.697*	-0.002<br>*p=0.982*
**oxyben-zoneC**	0.147<br>*p=0.152*	0.105<br>*p=0.308*	0.032<br>*p=0.759*	0.084<br>*p=0.416*	0.145<br>*p=0.159*	0.111<br>*p=0.282*	0.097<br>*p=0.346*	0.129<br>*p=0.212*	0.039<br>*p=0.7090*	**0.295**<br>***p=0.004***	0.13<br>*p=0.206*	-0.088<br>*p=0.396*	0.024<br>*p=0.8135*	1	0.159<br>*p=0.121*	0.105<br>*p=0.308*	0.171<br>*p=0.095*	**0.526**<br>***p<0.0001***	-0.017<br>*p=0.871*	**0.546**<br>***p<0.0001***	0.043<br>*p=0.680*	0.184<br>*p=0.073*	0.09<br>*p=0.383*
**BP**	0.114<br>*p=0.270*	0.042<br>*p=0.688*	0.036<br>*p=0.724*	0.003<br>*p=0.975*	0.11<br>*p=0.284*	-0.119<br>*p=0.249*	**0.252**<br>***p=0.013***	**0.536**<br>***p<0.0001***	**0.365**<br>***p=0.0002***	**0.34**<br>***p=0.001***	-0.012<br>*p=0.906*	0.115<br>*p=0.260*	0.102<br>*p=0.322*	0.159<br>*p=0.121*	1	0.043<br>*p=0.676*	0.087<br>*p=0.394*	0.122<br>*p=0.238*	**0.313**<br>***p=0.002***	-0.002<br>*p=0.986*	-0.028<br>*p=0.7890*	0.072<br>*p=0.483*	0.112<br>*p=0.276*
**daidzein**	0.122<br>*p=0.238*	**0.205**<br>***p=0.045***	0.148<br>*p=0.149*	0.195<br>*p=0.055*	**0.221**<br>***p=0.030***	0.133<br>*p=0.198*	0.118<br>*p=0.254*	0.104<br>*p=0.312*	0.046<br>*p=0.655*	0.004<br>*p=0.972*	**0.333**<br>***p=0.0009***	0.048<br>*p=0.637*	**0.44**<br>***p<0.0001***	0.105<br>*p=0.308*	0.043<br>*p=0.676*	1	**0.434**<br>***p<0.0001***	0.043<br>*p=0.677*	0.04<br>*p=0.695*	0.041<br>*p=0.692*	**0.274**<br>***p=0.007***	0.048<br>*p=0.641*	-0.113<br>*p=0.271*
**genistein**	0.194<br>*p=0.059*	0.098<br>*p=0.343*	**0.233**<br>***p=0.022***	**0.25**<br>***p=0.014***	**0.226**<br>***p=0.026***	0.115<br>*p=0.264*	0.009<br>*p=0.928*	**0.235**<br>***p=0.021***	0.032<br>*p=0.758*	0.028<br>*p=0.789*	0.148<br>*p=0.149*	-0.112<br>*p=0.273*	**0.279**<br>***p=0.006***	0.171<br>*p=0.095*	0.087<br>*p=0.394*	**0.434**<br>***p<0.0001***	1	**0.262**<br>***p=0.0100***	-0.031<br>*p=0.766*	0.122<br>*p=0.236*	0.184<br>*p=0.072*	0.078<br>*p=0.447*	-0.081<br>*p=0.432*
**BPAFC**	0.119<br>*p=0.250*	0.022<br>*p=0.834*	0.032<br>*p=0.760*	0.134<br>*p=0.195*	0.011<br>*p=0.917*	0.017<br>*p=0.872*	0.085<br>*p=0.410*	**0.234**<br>***p=0.022***	0.184<br>*p=0.073*	**0.268**<br>***p=0.008***	0.057<br>*p=0.582*	0.025<br>*p=0.811*	-0.061<br>*p=0.552*	**0.526**<br>***p<0.0001***	0.122<br>*p=0.238*	0.043<br>*p=0.677*	**0.262**<br>***p=0.0100***	1	0.085<br>*p=0.408*	**0.259**<br>***p=0.011***	0.03<br>*p=0.767*	0.19<br>*p=0.064*	0.005<br>*p=0.958*
**BPF**	**0.218**<br>***p=0.033***	0.117<br>*p=0.256*	0.038<br>*p=0.714*	0.006<br>*p=0.954*	0.003<br>*p=0.979*	0.09<br>*p=0.384*	**0.308**<br>***p=0.002***	**0.363**<br>***p=0.0003***	**0.279**<br>***p=0.006***	**0.242**<br>***p=0.017***	0.125<br>*p=0.223*	**0.233**<br>***p=0.021***	0.052<br>*p=0.617*	-0.017<br>*p=0.871*	**0.313**<br>***p=0.002***	0.04<br>*p=0.695*	-0.031<br>*p=0.766*	0.085<br>*p=0.408*	1	-0.039<br>*p=0.706*	-0.085<br>*p=0.410*	0.031<br>*p=0.767*	0.036<br>*p=0.728*
**BPC**	**0.265**<br>***p=0.009***	0.082<br>*p=0.428*	0.133<br>*p=0.196*	0.097<br>*p=0.348*	0.123<br>*p=0.234*	0.182<br>*p=0.076*	0.134<br>*p=0.193*	-0.09<br>*p=0.383*	-0.18<br>*p=0.080*	**0.226**<br>***p=0.027***	0.079<br>*p=0.442*	**-0.21**<br>***p=0.040***	0.046<br>*p=0.653*	**0.546**<br>***p<0.0001***	-0.002<br>*p=0.986*	0.041<br>*p=0.692*	0.122<br>*p=0.236*	**0.259**<br>***p=0.011***	-0.039<br>*p=0.706*	1	-0.124<br>*p=0.227*	0.016<br>*p=0.874*	-0.104<br>*p=0.315*
**SequolC**	-0.075<br>*p=0.465*	0.279<br>*p=0.006*	0.086<br>*p=0.403*	0.098<br>*p=0.342*	0.158<br>*p=0.123*	0.032<br>*p=0.756*	0.086<br>*p=0.404*	-0.012<br>*p=0.909*	0.166<br>*p=0.107*	-0.123<br>*p=0.231*	0.186<br>*p=0.069*	**0.287**<br>***p=0.005***	**0.337**<br>***p=0.001***	0.043<br>*p=0.680*	-0.028<br>*p=0.790*	**0.274**<br>***p=0.007***	0.184<br>*p=0.072*	0.03<br>*p=0.769*	-0.085<br>*p=0.410*	-0.124<br>*p=0.227*	1	0.008<br>*p=0.940*	0.125<br>*p=0.224*
**BPFC**	0.055<br>*p=0.592*	0.048<br>*p=0.644*	-0.064<br>*p=0.536*	-0.015<br>*p=0.886*	0.096<br>*p=0.350*	0.049<br>*p=0.636*	0.166<br>*p=0.106*	**0.346**<br>***p=0.001***	0.103<br>*p=0.316*	0.144<br>*p=0.162*	**0.247**<br>***p=0.015***	**0.169**<br>***p=0.100***	0.04<br>*p=0.697*	0.184<br>*p=0.073*	0.072<br>*p=0.483*	0.048<br>*p=0.641*	0.078<br>*p=0.447*	0.19<br>*p=0.064*	0.031<br>*p=0.7670*	0.016<br>*p=0.874*	0.008<br>*p=0.940*	1	0.037<br>*p=0.723*
**oxyben-zone**	0.014<br>*p=0.894*	0.117<br>*p=0.258*	0.047<br>*p=0.648*	0.067<br>*p=0.517*	0.006<br>*p=0.953*	-0.024<br>*p=0.814*	0.072<br>*p=0.488*	0.15<br>*p=0.144*	**0.341**<br>***p=0.001***	**0.265**<br>***p=0.009***	0.026<br>*p=0.800*	**0.218**<br>***p=0.032***	-0.002<br>*p=0.982*	0.09<br>*p=0.383*	0.112<br>*p=0.276*	-0.113<br>*p=0.271*	-0.081<br>*p=0.432*	0.005<br>*p=0.958*	0.036<br>*p=0.728*	-0.104<br>*p=0.315*	0.125<br>*p=0.224*	0.037<br>*p=0.723*	1

Abbreviations: MP, Methylparaben; EP, Ethylparaben; PP, Propylparaben; BP, Butylparaben; benzylP, Benzylparaben; BPA, Bisphenol A; BPS, Bisphenol S; BPF, Bisphenol F; BPAF, Bisphenol AF

Table S6. Medians and interquartile ranges (IQR) of steroid hormones in cancerous tissue by pathological ISUP grade (pISUP), with multiple comparisons across post-surgical ISUP grades and Gleason scores (pGS).

Intratumoral steroid levels (ng/g)	pISUP GG 1 (n=9)	pISUP GG 2 (n=61)	pISUP GG 3 (n=15)	pISUP GG 4 (n=8)	pISUP GG 5 (n=7)	Multiple comparison	
						by pISUP (1-5)	by pGS (6–9)
Preg	1.893 (1.103; 3.182)	1.503 (0.733; 2.513)	1.893 (1.065; 4.203)	2.628 (1.203; 3.798)	3.26 (0.937; 4.272)	n.s.	n.s.
PregC	878.2 (538.4; 1015.1)	745.7 (622.8; 879.1)	682.2 (619.8; 1048.1)	532.6 (473.5; 806.0)	920.7 (628.9; 1252.3)	n.s.	n.s.
17OHPreg	1.547 (0.843; 2.153)	1.772 (0.377; 3.244)	1.326 (0.377; 4.419)	1.768 (0.766; 3.877)	2.351 (0.901; 3.594)	n.s.	n.s.
17OHPregC	25.65 (20.36; 32.20)	24.45 (18.28; 30.66)	25.20 (14.98; 34.08)	21.29 (17.31; 32.94)	27.52 (12.7; 30.98)	n.s.	n.s.
DHEA	2.41 (1.317; 9.904)	2.381 (0.377; 6.219)	1.21 (0.377; 8.022)	0.567 (0.377; 7.285)	5.572 (2.78; 8.022)	n.s.	n.s.
DHEAC	82.7 (50.0; 277.5)	84.8 (27.6; 135.5)	75.0 (20.6; 162.6)	39.36 (16.0; 95.9)	106.0 (17.2; 209.6)	n.s.	n.s.
7α-HydroxyDHEA	0.151 (0.151; 0.29)	0.151 (0.151; 0.31)	0.151 (0.151; 0.357)	0.151 (0.151; 0.353)	0.306 (0.151; 0.85)	n.s.	n.s.
7-ketoDHEA	0.112 (0.075; 0.173)	0.084 (0.019; 0.145)	0.056 (0.019; 0.295)	0.092 (0.019; 0.165)	0.178 (0.057; 0.25)	n.s.	n.s.
7-ketoDHEAC	0.47 (0.417; 0.642)	0.505 (0.317; 0.746)	0.385 (0.262; 0.633)	0.738 (0.672; 0.848)	0.642 (0.579; 0.939)	4>3,2	8>7
7β-HydroxyDHEA	0.189 (0.189; 0.189)	0.189 (0.189; 0.189)	0.189 (0.189; 0.189)	0.189 (0.189; 0.189)	0.189 (0.189; 0.189)	n.s.	n.s.
17OHProg	0.075 (0.075; 0.075)	0.075 (0.075; 0.075)	0.075 (0.075; 0.075)	0.075 (0.075; 0.075)	0.075 (0.075; 0.075)	n.d.	n.d.
17OHProgC	12.57 (10.14; 21.96)	13.01 (8.89; 16.42)	11.36 (8.83; 15.01)	9.61 (7.00; 13.01)	13.73 (7.99; 19.74)	n.s.	n.s.
A4	0.019 (0.019; 0.019)	0.019 (0.019; 0.038)	0.019 (0.019; 0.032)	0.019 (0.019; 0.04)	0.05 (0.019; 0.15)	5>1	9>6
T	0.031 (0.014; 0.052)	0.032 (0.015; 0.052)	0.043 (0.027; 0.117)	0.025 (0.012; 0.083)	0.049 (0.023; 0.142)	n.s.	n.s.
TC	0.226 (0.108; 0.396)	0.124 (0.046; 0.207)	0.128 (0.087; 0.416)	0.154 (0.082; 0.227)	0.134 (0.079; 0.189)	3>2	n.s.
11KT	0.075 (0.075; 0.075)	0.075 (0.075; 0.075)	0.075 (0.075; 0.075)	0.075 (0.075; 0.075)	0.075 (0.075; 0.075)	n.s.	9>7
11OHT	0.038 (0.038; 0.061)	0.038 (0.038; 0.038)	0.038 (0.038; 0.038)	0.038 (0.038; 0.041)	0.038 (0.038; 0.038)	1>2,3	6>7
DHT	0.829 (0.584; 2.309)	0.751 (0.525; 1.25)	0.79 (0.597; 1.475)	0.953 (0.458; 1.317)	0.787 (0.57; 1.291)	n.s.	n.s.
DHTC	1.055 (0.664; 1.374)	0.893 (0.629; 1.176)	0.811 (0.668; 1.422)	0.603 (0.375; 1.27)	0.58 (0.292; 1.055)	n.s.	n.s.
E1	0.028 (0.028; 0.035)	0.028 (0.028; 0.052)	0.028 (0.028; 0.046)	0.028 (0.028; 0.046)	0.043 (0.028; 0.05)	n.s.	n.s.
E1C	1.643 (1.025; 2.129)	1.504 (1.223; 1.982)	1.363 (1.025; 1.808)	1.099 (0.841; 1.507)	1.79 (1.73; 2.681)	5,2,1>4	9,7,6>8
E2	0.032 (0.032; 0.032)	0.032 (0.032; 0.032)	0.032 (0.032; 0.032)	0.032 (0.032; 0.032)	0.032 (0.032; 0.032)	n.s.	n.s.
E2C	0.171 (0.096; 0.197)	0.169 (0.096; 0.57)	0.156 (0.073; 0.719)	0.174 (0.099; 0.405)	0.086 (0.032; 0.176)	2>5	7>9
E3	0.028 (0.028; 0.028)	0.028 (0.028; 0.028)	0.028 (0.028; 0.028)	0.028 (0.028; 0.037)	0.028 (0.028; 0.028)	n.s.	n.s.
E3C	2.031 (1.655; 2.282)	2.109 (1.88; 2.763)	2.034 (1.568; 2.538)	2.144 (1.742; 2.361)	2.343 (1.998; 2.772)	n.s.	n.s.
DHP	0.019 (0.019; 0.117)	0.039 (0.019; 0.087)	0.019 (0.019; 0.06)	0.097 (0.029; 0.115)	0.056 (0.036; 0.198)	5,4>3	n.s.
F	4.62 (3.362; 14.48)	4.75 (2.27; 12.15)	4.23 (2.98; 17.71)	5.81 (3.45; 13.38)	17.61 (4.70; 35.38)	5>2	n.s.
E	2.33 (0.81; 6.84)	2.31 (1.31; 5.84)	3.77 (0.75; 9.27)	4.28 (2.20; 9.19)	22.40 (1.59; 48.38)	5>3,2,1	9>6,7
B	0.144 (0.057; 0.31)	0.089 (0.057; 0.212)	0.057 (0.057; 0.256)	0.196 (0.095; 0.398)	0.362 (0.057; 0.446)	n.s.	n.s.
BC	0.748 (0.282; 3.984)	0.393 (0.132; 4.234)	1.268 (0.158; 4.428)	0.501 (0.133; 4.924)	0.344 (0.167; 7.084)	n.s.	n.s.
21DOF	0.019 (0.019; 0.033)	0.019 (0.019; 0.043)	0.029 (0.019; 0.044)	0.019 (0.019; 0.04)	0.028 (0.019; 0.071)	n.s.	9>8
21DOFC	10.35 (5.90; 12.24)	8.74 (5.53; 12.55)	9.2 (6.58; 12.02)	9.79 (4.76; 12.55)	7.49 (6.18; 19.07)	n.s.	n.s.

n.s. – not significant; groups did not significantly differ from each other (α = 0.05)
n.d. – not determined; analyte levels were below the lower limit of quantification (LLOQ) in the majority of samples; analyses were not performed.

Abbreviations: C after steroid name indicates a conjugated form; Preg, Pregnenolone; 17OHPreg, 17-Hydroxypregnenolone; DHEA, Dehydroepiandrosterone; 7α-HydroxyDHEA, 7α-Hydroxydehydroepiandrosterone; 7-ketoDHEA, 7-ketodehydroepiandrosterone; 7β-HydroxyDHEA, 7β-Hydroxydehydroepiandrosterone; 17OHProg, 17-Hydroxyprogesterone; A4, Androstenedione; T, Testosterone; 11KT, 11-ketotestosterone; 11OHT, 11β-Hydroxytestosterone; DHT, 5α-Dihydrotestosterone; E1, Estrone; E2, Estradiol; E3, Estriol; DHP, 5α-Dihydroprogesterone; F, Cortisol; E, Cortisone; B, Corticosterone; 21DOF, 21-Deoxycortisol.

Table S7 Medians and interquartile ranges (IQR) of EDCs in cancerous tissue by post-surgical ISUP grade (pISUP), with multiple comparisons across post-surgical ISUP grades and Gleason scores (pGS).

Intratumoral ED levels (ng/g)	pISUP 1 (n=9)	pISUP 2 (n=61)	pISUP 3 (n=15)	pISUP 4 (n=8)	pISUP 5 (n=7)	Multiple comparison	
						by pISUP (1-5)	by pGS (6–9)
MP	0.673 (0.211; 7.554)	0.721 (0.184; 2.663)	0.518 (0.237; 0.966)	0.225 (0.151; 3.91)	0.296 (0.269; 0.507)	n.s.	n.s.
MPC	73.46 (40.81; 335.46)	59.32 (29.96; 129.63)	78.58 (54.28; 144.00)	62.62 (43.34; 139.47)	39.81 (33.08; 61.33)	n.s.	n.s.
EP	0.05 (0.028; 1.311)	0.051 (0.028; 0.314)	0.057 (0.028; 0.08)	0.028 (0.028; 0.229)	0.028 (0.028; 0.07)	n.s.	n.s.
EPC	0.976 (0.458; 2.772)	0.45 (0.328; 0.939)	0.646 (0.361; 1.023)	0.467 (0.226; 0.987)	0.52 (0.33; 0.845)	1>2,4	6>7
PP	1.082 (0.228; 9.176)	0.909 (0.262; 2.898)	0.713 (0.528; 1.458)	0.496 (0.158; 1.458)	0.398 (0.135; 0.988)	n.s.	n.s.
PPC	4.2 (2.92; 18.27)	3.529 (2.102; 5.868)	4.168 (2.775; 5.727)	3.83 (2.028; 5.998)	3.399 (1.79; 4.232)	n.s.	n.s.
BP	0.038 (0.038; 0.038)	0.038 (0.038; 0.038)	0.038 (0.038; 0.038)	0.038 (0.038; 0.062)	0.038 (0.038; 0.038)	4>3,1	n.s.
BPC	0.047 (0.038; 0.061)	0.12 (0.038; 0.246)	0.163 (0.038; 0.384)	0.108 (0.038; 0.363)	0.116 (0.088; 0.381)	5,3>1	9>6
benzylP	0.028 (0.028; 0.061)	0.028 (0.028; 0.028)	0.028 (0.028; 0.028)	0.028 (0.028; 0.035)	0.028 (0.028; 0.047)	n.s.	n.s.
BPA	0.605 (0.151; 2.921)	0.287 (0.151; 0.55)	0.151 (0.151; 0.318)	0.202 (0.151; 0.634)	0.183 (0.151; 1.124)	n.s.	n.s.
BPAC	1.379 (1.225; 3.208)	1.874 (1.15; 2.764)	1.723 (1.043; 2.377)	0.942 (0.66; 2.214)	1.642 (0.805; 6.415)	n.s.	n.s.
BPS	1.336 (0.171; 1.498)	0.315 (0.144; 0.672)	0.366 (0.075; 0.512)	0.173 (0.075; 0.593)	0.255 (0.238; 1.426)	n.s.	n.s.
BPSC	0.54 (0.283; 0.895)	0.353 (0.146; 0.669)	0.223 (0.137; 0.617)	0.142 (0.075; 0.57)	0.182 (0.075; 1.719)	n.s.	n.s.
BPF	0.038 (0.038; 0.038)	0.038 (0.038; 0.038)	0.038 (0.038; 0.038)	0.038 (0.038; 0.05)	0.038 (0.038; 0.038)	n.d.	n.d.
BPFC	0.055 (0.038; 0.127)	0.058 (0.038; 0.102)	0.072 (0.038; 0.119)	0.038 (0.038; 0.086)	0.038 (0.038; 0.065)	n.s.	n.s.
BPAFC	0.529 (0.374; 0.729)	0.594 (0.504; 0.79)	0.643 (0.283; 0.833)	0.661 (0.46; 0.8)	0.582 (0.463; 1.388)	n.s.	n.s.
oxybenzone	0.057 (0.057; 0.116)	0.057 (0.057; 0.069)	0.057 (0.057; 0.089)	0.057 (0.057; 0.081)	0.108 (0.057; 0.272)	3>1,2,4	9>8
oxybenzoneC	3.362 (2.171; 5.902)	6.405 (3.755; 9.201)	5.335 (3.21; 11.976)	5.147 (1.67; 8.576)	7.118 (4.325; 7.519)	n.s.	n.s.
daidzein	0.038 (0.038; 0.321)	0.038 (0.038; 0.038)	0.038 (0.038; 0.038)	0.038 (0.038; 0.038)	0.038 (0.038; 0.038)	n.s.	n.s.
daidzeinC	0.038 (0.038; 1.005)	0.038 (0.038; 0.105)	0.038 (0.038; 0.217)	0.052 (0.038; 0.203)	0.065 (0.038; 0.35)	n.s.	n.s.
genistein	0.038 (0.038; 0.064)	0.038 (0.038; 0.038)	0.038 (0.038; 0.038)	0.038 (0.038; 0.038)	0.038 (0.038; 0.038)	n.d.	n.d.
genisteinC	0.19 (0.063; 0.519)	0.038 (0.038; 0.14)	0.194 (0.038; 0.695)	0.071 (0.038; 0.223)	0.038 (0.038; 0.682)	1>2	n.s.

n.s. – not significant; groups did not significantly differ from each other (α = 0.05)

n.d. – not determined; analyte levels were below the lower limit of quantification (LLOQ) in the majority of samples; analyses were not performed.

Abbreviations: C after EDC name indicates a conjugated form; MP, Methylparaben; EP, Ethylparaben; PP, Propylparaben; BP, Butylparaben; benzylP, Benzylparaben; BPA, Bisphenol A; BPS, Bisphenol S; BPF, Bisphenol F; BPAF, Bisphenol AF.

References

1 Vitku, J., Horackova, L., Kolatorova, L. *et al.* Derivatized versus non-derivatized LC-MS/MS techniques for the analysis of estrogens and estrogen-like endocrine disruptors in human plasma. *Ecotoxicology and Environmental Safety* 2023 **260**.

2 Simkova, M., Kolatorova, L., Drasar, P. *et al.* An LC-MS/MS method for the simultaneous quantification of 32 steroids in human plasma. *Journal of Chromatography B-Analytical Technologies in the Biomedical and Life Sciences* 2022 **1201**.

3 Vitku, J., Skodova, T., Varausova, A. *et al.* Endocrine Disruptors and Estrogens in Human Prostatic Tissue. *Physiol Res* 2023 **72** S411-s422.

4 Kolatorova Sosvorova, L., Chlupacova, T., Vitku, J. *et al.* Determination of selected bisphenols, parabens and estrogens in human plasma using LC-MS/MS. *Talanta* 2017 **174** 21-28.

5 Skodova, T., Vitku, J., Bradac, O. *et al.* LC-MS/MS techniques for the analysis of steroid panel in human cerebrospinal fluid. *Neurochem Int* 2025 **191** 106080.

6 Europen Medicines Agency, *ICH guideline M10 on bioanalytical method validation and study sample analysis*, 2023. https://www.ema.europa.eu/en/documents/scientific-guideline/ich-guideline-m10-bioanalytical-method-validation-step-5_en.pdf. Accessed: 21.2.2026
